# Non-line-of-sight reconstruction with signal–object collaborative regularization

**DOI:** 10.1038/s41377-021-00633-3

**Published:** 2021-09-24

**Authors:** Xintong Liu, Jianyu Wang, Zhupeng Li, Zuoqiang Shi, Xing Fu, Lingyun Qiu

**Affiliations:** 1 Yau Mathematical Sciences Center, Tsinghua University, 100084 Beijing, China; 2 State Key Laboratory of Precision Measurement Technology and Instruments, Department of Precision Instrument, Tsinghua University, 100084 Beijing, China; 3 Key Laboratory of Photonic Control Technology (Tsinghua University), Ministry of Education, 100084 Beijing, China; 4 Department of Mathematical Sciences, Tsinghua University, 100084 Beijing, China; 5 Yanqi Lake Beijing Institute of Mathematical Sciences and Applications, 101408 Beijing, China

**Keywords:** Imaging and sensing, Single photons and quantum effects

## Abstract

Non-line-of-sight imaging aims at recovering obscured objects from multiple scattered lights. It has recently received widespread attention due to its potential applications, such as autonomous driving, rescue operations, and remote sensing. However, in cases with high measurement noise, obtaining high-quality reconstructions remains a challenging task. In this work, we establish a unified regularization framework, which can be tailored for different scenarios, including indoor and outdoor scenes with substantial background noise under both confocal and non-confocal settings. The proposed regularization framework incorporates sparseness and non-local self-similarity of the hidden objects as well as the smoothness of the signals. We show that the estimated signals, albedo, and surface normal of the hidden objects can be reconstructed robustly even with high measurement noise under the proposed framework. Reconstruction results on synthetic and experimental data show that our approach recovers the hidden objects faithfully and outperforms state-of-the-art reconstruction algorithms in terms of both quantitative criteria and visual quality.

## Abstract (web summary)

A Non-line-of-sight reconstruction framework with collaborative regularization of the hidden scene and the signal is proposed to obtain albedo and surface normal of the targets with robustness to noise.

## Graphical abstract

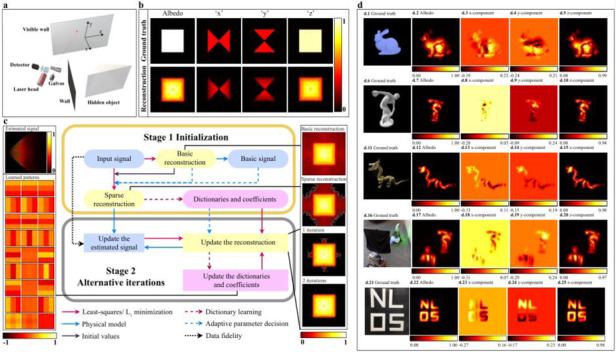

## Introduction

**Fig. 1:**
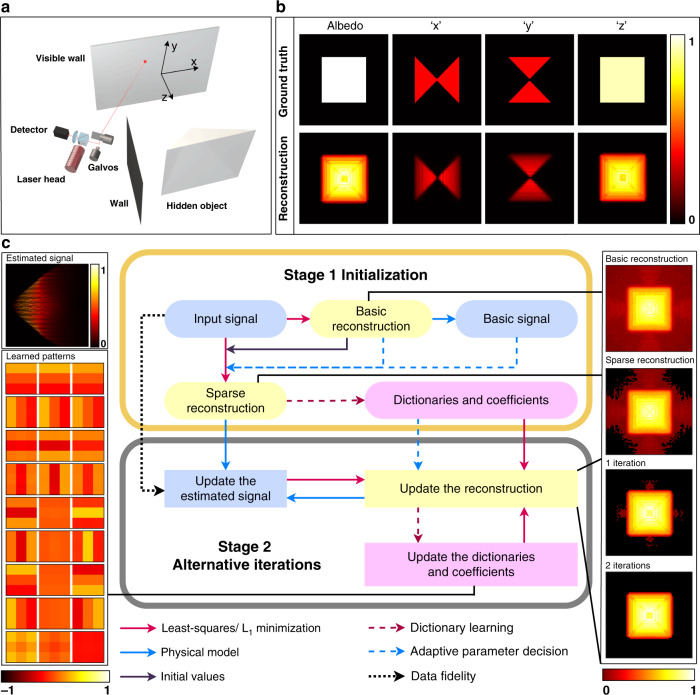
Schematic of NLOS layout and the proposed SOCR reconstruction algorithm. **a** To reconstruct the unseen object, some light sources are used to illuminate a visible wall. The photons bounced back from the object are detected at several points on the visible wall. **b** Ground truth and our reconstruction. The *x*, *y*, and *z* components are displayed in their absolute values. **c** Flowchart of the proposed framework. The estimated signal and the learned patterns of the target are shown on the left. Reconstructed albedo is shown on the right

Non-line-of-sight (NLOS) imaging focuses on recovering objects that are hidden from the direct line of sight. In real applications, lasers or other light sources are used to illuminate a visible wall, the scattered light from which reaches the hidden object and is scattered back again. The photons collected by detectors such as single photon avalanche diode (SPAD) or conventional cameras can be used to recover the location, shape, albedo, and normal of the target. This problem has attracted much attention recently due to its potential applications such as auto-driving, survivor-rescuing, and remote sensing. A typical schematic of the NLOS layout is shown in Fig. 1a.

The NLOS reconstruction problem belongs to the inverse problem in mathematics, aiming to find the hidden scene that matches the detected signal. This problem is usually ill-posed due to measurement noise, depth and scale ambiguity, and non-uniqueness of the solution^1^.

The study in NLOS dates back to Velten et al. ^2^ in 2012 when the back-projection method was proposed. After that, the widely used confocal experimental settings were designed by O’Toole et al. ^3^ Geometric-based approaches^4,5^ use only the time of flight to reconstruct the hidden target. Instead of treating light as rays, NLOS can also be formulated as the propagation of a wave^6–9^. With the development of deep learning, neural-network-based NLOS reconstruction methods are emerging^10–14^. Many different experimental settings and algorithms are designed to improve practicability^15–23^.

Several efficient NLOS imaging algorithms in confocal settings have been proposed. The light-cone-transform^3^ and frequency-wavenumber migration methods^6^ (F-K) reconstruct the albedo of the hidden target in a time-efficient way using the fast Fourier transform. The directional light-cone-transform^24^ (D-LCT) reconstructs the albedo and surface normal simultaneously. The algorithm proposed by Heide et al. ^25^ considers partially occluded scene and reconstructs both the albedo and surface normal, at a rather high computational cost and memory usage, though. Note that these methods for confocal settings do not generalize directly to non-confocal scenarios, the more general version of NLOS measurement. In real applications, confocal experiments are harder to implement due to the beam interference from illuminating and detecting the same location on the visible wall^6^. Although non-confocal measurements can be converted to confocal ones based on the normal moveout correction technique^26^, the reconstruction results using the converted measurements usually contain a lot of artifacts due to the approximation error, especially in the case of the large interval between illumination and detection positions. In the non-confocal case, the Laplacian of Gaussian filtered back-projection^27^ (LOG-BP) and the phasor field methods^7,8^ reconstruct the albedo efficiently without providing surface normal.

Measurement noise is one of the major obstacles to get high-quality reconstructions in NLOS inverse problems. When the measurement noise is high, the targets reconstructed are usually noisy with blurred boundaries. Several methods have been developed to improve the quality of the reconstruction. The back-projection algorithm can be enhanced by a post-processing step using the Laplacian of Gaussian filter^27^ or introducing weighting factors^28^. A wide class of approaches solves optimization problems with regularization terms of the hidden object^20,24,25,29–32^. The light-cone-transform can be improved by introducing L_1_ and TV regularizations^3^ (LCT + L_1_ + TV). The D-LCT algorithm uses the L_2_ regularization term to overcome the rank deficiency. Besides, it is possible to attenuate the noise in the measurements as a preprocessing step. However, the pre-existing denoising techniques^33–35^ tend to over smooth the measured signal and lead to reconstructions with less fine structures. An example is provided in Section 1 of the Supplement.

In this paper, we propose an NLOS reconstruction framework with collaborative regularization of the signal and the reconstructed object, which we term the signal–object collaborative regularization (SOCR) method. Instead of using the measurement directly, we introduce an approximation of the oracle signal and treat it as an optimization variable. We focus on the sparseness and non-local self-similarity of the hidden object as well as the smoothness of the estimated signal. A joint prior term for NLOS imaging is constructed, which is a combination of three different priors. We simplify the physical model proposed by Tsai et al. ^31^ as a linear model and reconstruct the hidden scene by solving a least-squares problem with collaborative regularization. The main steps of the algorithm are shown in Fig. 1c. To the best of our knowledge, this is the first work that introduces the approximation of oracle signals and the signal–object collaborative regularization framework in NLOS imaging. The proposed framework is powerful in reconstructing both the albedo and the surface normal of the hidden targets under the general non-confocal settings, and the physical model used reduces to the directional albedo model proposed by Young et al. ^24^ for the special case of the confocal settings. The proposed method reconstructs the targets faithfully with clear local structures and sharp boundaries, outperforming previous methods in terms of both quantitative criteria and visual quality (see Table 1).

**Table 1:** Comparisons of voxel-based NLOS reconstruction algorithms

Methods	Confocal		Non-confocal		Prior	Noise robustness
	Albedo	Normal	Albedo	Normal		
LOG-BP^27^	✓	✗	✓	✗	Object	Low
LCT + L_1_ + TV^3^	✓	✗	✗	✗	Object	Medium
Occluder^25^	✓	✓	✗	✗	Object	Not known
F-K^6^	✓	✗	✗	✗	None	Medium
D-LCT^24^	✓	✓	✗	✗	Object	Medium high
Phasor Field^8^	✓	✗	✓	✗	None	Medium
SOCR	✓	✓	✓	✓	Signal & object	High

The proposed method is the only one that is capable of reconstructing both albedo and surface normal in both confocal and non-confocal settings. It is also the only one that incorporates the priors of the signal and object with the highest robustness to measurement noise

**Table 2:** Comparisons of the D-LCT and SOCR reconstructions of the pyramid

Methods	Excessive voxels	Depth error (RMSE) (cm)	Normal error (mean)	Normal error (maximum)
D-LCT	426	1.10	2.90°	11.81°
SOCR	65	0.65	1.62°	5.23°

## Results

We demonstrate the effectiveness of the proposed framework with both synthetic and experimental data. The results are compared with the Laplacian of Gaussian filtered back-projection^27^ (LOG-BP), L_1_ and TV regularized light-cone-transform^3^ (LCT + L_1_ + TV), frequency-wavenumber migration^6^ (F-K), and directional light-cone-transform^24^ (D-LCT) methods. Note that the LOG-BP, LCT + L_1_ + TV, and F-K methods can only recover the albedo of the hidden scene, while D-LCT can recover both the albedo and surface normal simultaneously.

### Synthetic data

The Zaragoza NLOS synthetic dataset^36^ is a public dataset containing synthetic data rendered from several hidden objects. For confocal experiments, we choose the letter T, US Air Force (USAF) test resolution chart, and Stanford bunny from this dataset as typical examples of a simple plane object, a plane target of several disjoint components, and a surface with complex structures, respectively. All these three objects are 0.5 m from the diffuse wall. For the letter T and the Stanford bunny, the wall in the line of sight is sampled at 64 × 64 points over a region of 0.6 × 0.6 m^2^ and the photon travel distance is 0.0025 m in each time bin. For the instance of USAF, the illumination points are downsampled to 64 × 64 grids over a region of 1 × 1 m^2^ and the photon travels 0.003 m in each time bin.

**Fig. 2:**
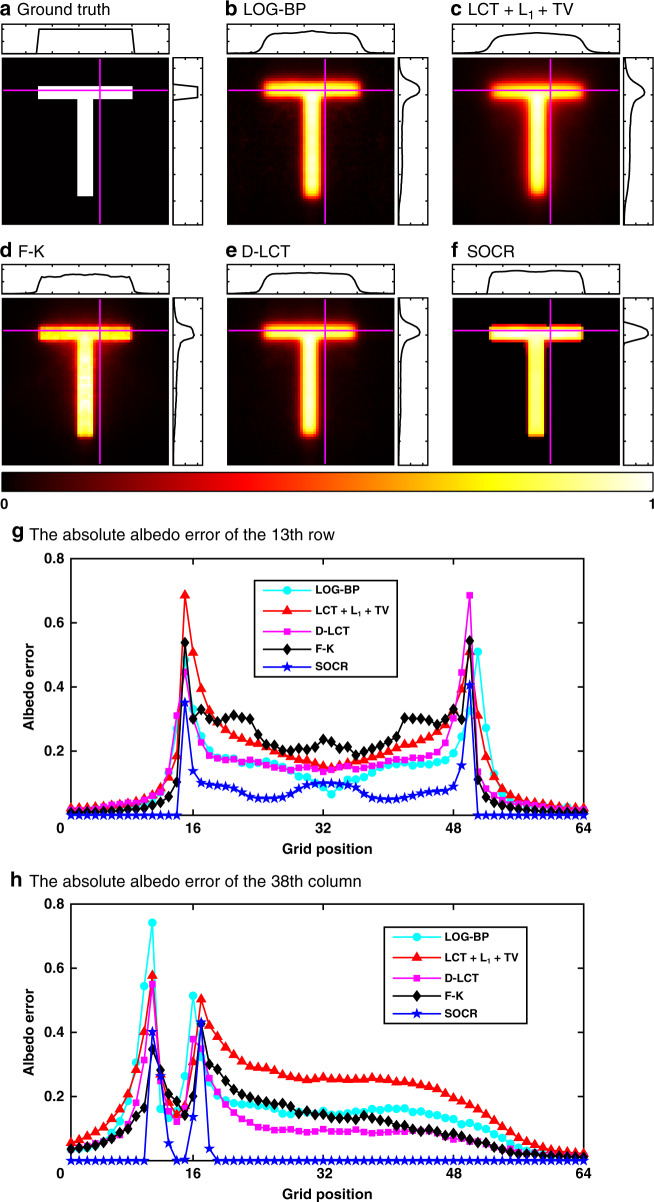
Reconstruction results of the letter T (confocal). The ground truth is shown in **a**. Reconstructed albedo is shown in **b**–**f**. The absolute albedo error of two cross-section lines (the 13th row and 38th column) are shown in **g** and **h**. The proposed method has the smallest error

The reconstruction results of the letter T are shown in Fig. 2b–f. Maximum intensity projections along the depth direction are shown in the hot colormap. In addition, two cross-section lines with the albedo values of the 13th row and 38th column are shown on the top and right panels in each sub-figure. It is shown that all methods find the letter T correctly. The root mean square error (RMSE) of our reconstructed albedo is 0.0788, which is much smaller than those obtained by LOG-BP (0.1489), LCT + L_1_ + TV (0.1547), F-K (0.1079), and D-LCT (0.1298) methods. By thresholding the albedo values <0.55, our reconstruction matches perfectly with the ground truth, with RMSE further reducing to 0.0572, much less than that of the D-LCT algorithm (0.1266). Furthermore, we compare in Fig. 2g and h the absolute error of the albedo along these two cross-section lines. It is shown that our reconstruction has the smallest error.

**Fig. 3:**
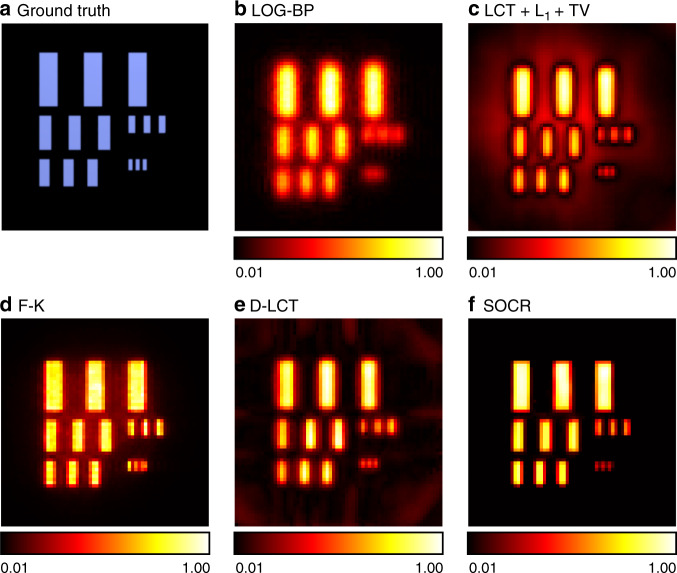
Reconstruction results of the USAF (confocal). The ground truth is shown in **a**. Reconstructed albedo is shown in **b**-**f**. The proposed algorithm reconstructs the object with the best visual quality

In Fig. 3, we compare the depth maps of the reconstructed target USAF. The background of LOG-BP, LCT + L_1_ + TV, F-K, and the D-LCT reconstructions are not clean, while the SOCR reconstruction matches very well with the ground truth.

**Fig. 4:**
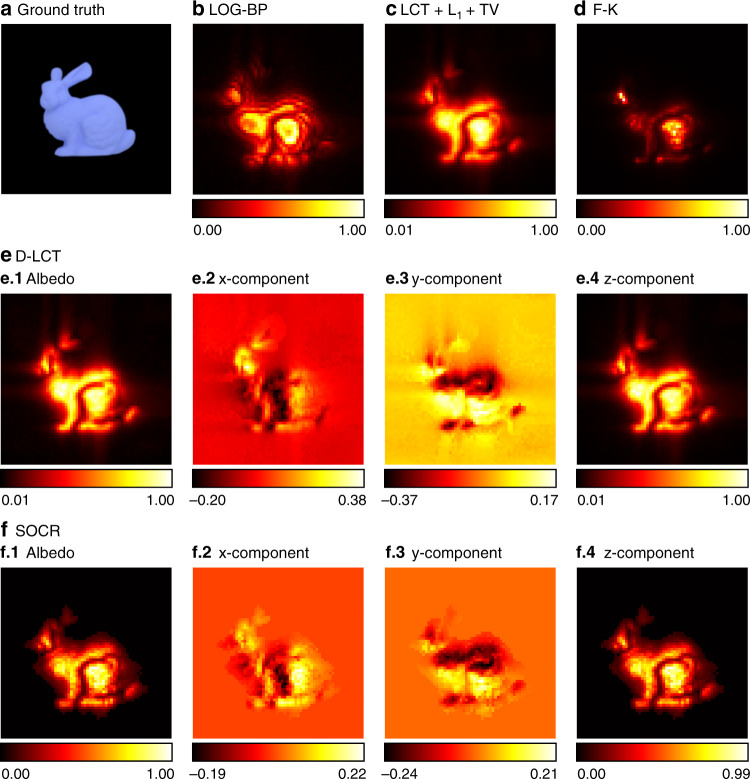
Reconstruction results of the Stanford bunny (confocal). The ground truth is shown in **a**. Reconstructions are shown in **b–f**. The proposed method provides a sparser reconstruction than the D-LCT algorithm, and the boundary is much sharper than the one given by LCT + L_1_ + TV

The reconstruction results of the Stanford bunny are compared in Fig. 4. The LOG-BP algorithm only finds the location of the target approximately and the F-K algorithm fails to recover the ears of the bunny. Although the LCT + L_1_ + TV algorithm recovers the albedo correctly, the boundary of the reconstructed target is blurry. Both our method and the D-LCT method reconstruct the hidden object well, while our model provides a sharper boundary of the hidden target in each of the three components.

**Fig. 5:**
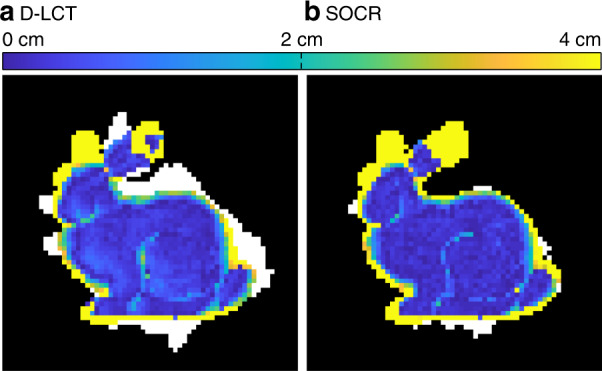
Absolute depth error of the D-LCT and the SOCR reconstruction. The absolute depth error of the D-LCT reconstruction is shown in **a**. The absolute depth error of the SOCR reconstruction is shown in **b**. The SOCR reconstruction has a smaller absolute depth error at the chest and legs of the bunny. The D-LCT reconstruction has 254 excessive voxels outside the ground truth, which is 20.63% of the non-zero voxels contained in the ground truth (1231), while the proposed algorithm has 46 excessive voxels (3.74%), which is about one-sixth of the D-LCT reconstruction

In Fig. 5 we compare the depth error of the D-LCT and SOCR reconstructions. Albedo values that are smaller than 7% of the maximum intensity are thresholded to zero. The background is shown in black, while the reconstruction outside the ground truth is shown in white. In this experiment, the oracle scene contains 1231 non-zero albedo values. The boundary of our reconstruction matches the ground truth better with only 46 voxels outside the ground truth, which is about one-sixth of the D-LCT reconstruction (254 voxels). In addition, the depth error of our reconstruction at the legs and chest of the bunny is also smaller.

To demonstrate the efficiency of our algorithm in recovering the surface normal, we generate synthetic data of a pyramid (see Fig. 1a), with a simplified version of the three-point rendering model^37^ under the confocal settings. The central axis of the pyramid is vertical to the visible wall and it is 0.2 m in height with a base length of 0.5 m. The wall in the line of sight is sampled at 64 × 64 points over a region of 2 × 2 m^2^ and the photon travel distance is 0.0096 m in each time bin. In Fig. 1b and c, we show the reconstruction, estimated signal, and learned patterns of the pyramid. The results are gradually improved as the iteration proceeds.

**Fig. 6:**
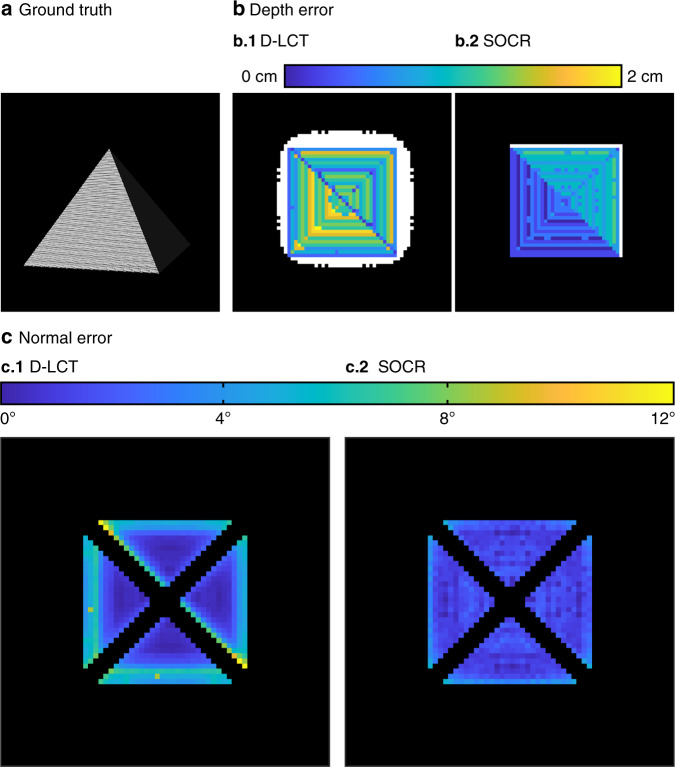
Absolute depth error and normal error of the pyramid (confocal). The ground truth is shown in **a**. Absolute depth error of the D-LCT and SOCR reconstructions is compared in **b**. Normal error of the D-LCT and SOCR reconstructions is shown in **c**. The depth error is shown in the same way as in Fig. 5, and the normal error is defined as the angle between the normal of the reconstruction and the ground truth

In Fig. 6b we compare the depth error of the D-LCT and SOCR reconstructions. Albedo values that are <15% of the maximum intensity are thresholded to zero. Reconstruction outside the ground truth is shown in white. The D-LCT reconstruction fails to capture the boundary correctly, with 426 excessive voxels outside the ground truth. In contrast, the proposed model provides an accurate estimation of the target, with only 65 excessive voxels. The depth RMSE of the SOCR reconstruction is 0.65 cm at the target, which is 41% smaller than the D-LCT reconstruction (1.10 cm).

In Fig. 6c we show the error of surface normal, which is defined as the angle between the reconstruction and the ground truth. The normal on the edges of the pyramid is not well defined and thus not included. Our algorithm provides an accurate estimation of the surface normal of the entire target, while the result of the D-LCT algorithm has a larger surface normal error near the edges. The mean normal error of D-LCT and our algorithm are 2.90° and 1.62°, respectively. Besides, the maximum normal error of the D-LCT algorithm is 11.81°, which is two times larger than ours (5.23°). Quantitative comparisons of the D-LCT and SOCR reconstructions are summarized in Tabel 2.

**Fig. 7:**
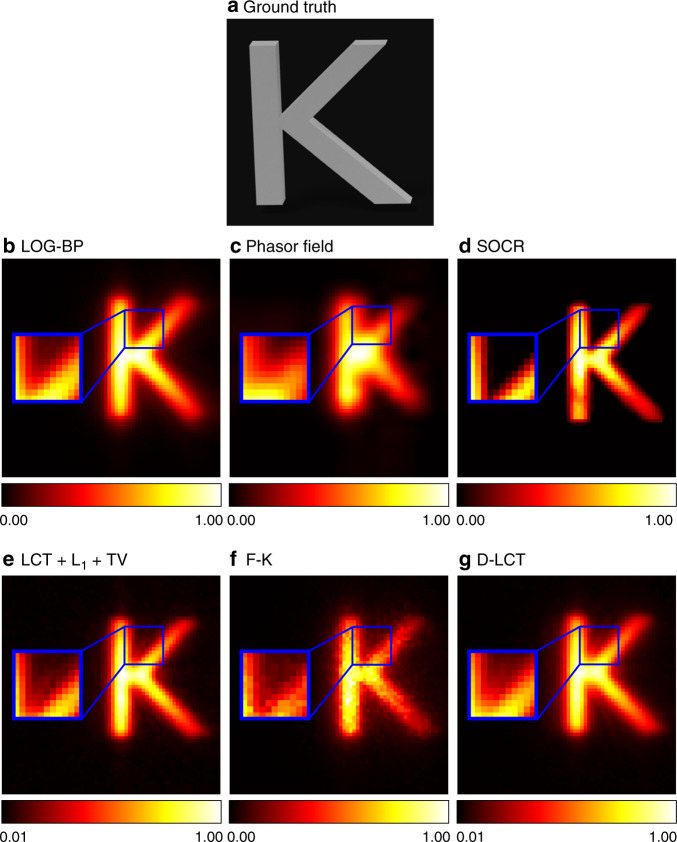
Reconstruction results of the letter K (non-confocal). The ground truth is shown in **a**. Reconstructions are shown in **b**–**g**. The F-K reconstruction is noisy. The LCT + L_1_ + TV and D-LCT reconstructions are blurry and contain artifacts. The phasor field method does not provide a clear reconstruction. The proposed method has the highest contrast and the corners are well reconstructed

To demonstrate the efficiency of our method under non-confocal settings, we compare our method with existing non-confocal solvers (the LOG-BP algorithm and the phasor field method^8^). Besides, we bring the confocal solvers (LCT + L_1_ + TV and F-K) into comparison by converting the non-confocal measurements to confocal data using the midpoint approximation technique^6^. We use the simulated data of the letter K from the NLoS Benchmark dataset^38^ to test the algorithms. The visible wall is illuminated at 64 × 64 points in a region of 0.512 × 0.512 m^2^. The detection point locates at the center of the illuminating region. The photon travel distance is 0.001 m per second. Reconstruction results are shown in Fig. 7. The phasor field method fails to reconstruct the details at this spatial resolution and the result of the LOG-BP is blurry. The reconstruction result of the F-K method is noisy. The LCT + L_1_ + TV and D-LCT methods introduce artifacts, which may arise from the approximation error in the confocal signals. The proposed method reconstructs the letter with the highest contrast and little noise. The blue box in each subfigure shows a zoom-in of a corner of the hidden target. In our reconstruction, the two strokes of the letter K are well separated, while all other methods provide blurry reconstructions.

### Measured data

**Fig. 8:**
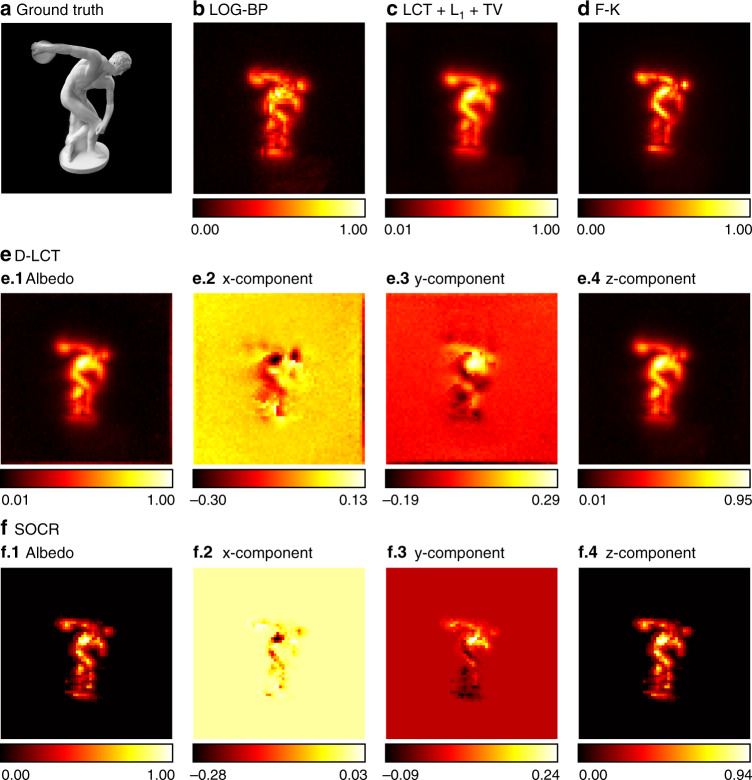
Reconstruction results of the statue (confocal, 10 min). The ground truth is shown in **a**. Reconstructions are shown in **b**–**f**. Our reconstruction has the highest contrast, with clear local structures and less noise outside the target. The three components of our reconstruction are jointly sparse due to the sparseness prior imposed on the albedo

We use the Stanford dataset^6^ to test our framework with measured data under confocal settings. The measurements are captured at 512 × 512 focal points over a square region of 2 × 2 m^2^ and downsampled to 64 × 64. The hidden scenes are 1 m from the illumination wall. For the instance of the statue, the exposure time is 10 min. As is shown in Fig. 8, the reconstructed albedo of our algorithm has higher contrast compared to other methods. Besides, the three components of our reconstruction are clear with less noise.

**Fig. 9:**
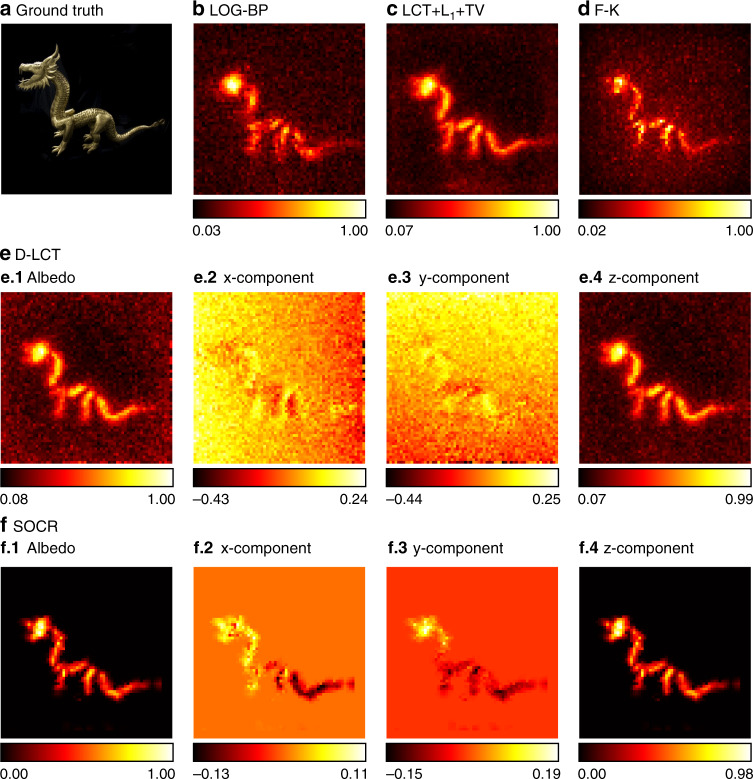
Reconstruction results of the dragon (confocal, 60 min). The ground truth is shown in **a**. Reconstructions are shown in **b**–**f**. The reconstruction results of the BP, LCT + L_1_ + TV, F-K, and D-LCT methods are noisy due to the specularity of the dragon and heavy noise in measured data. Our framework provides a reconstruction with little noise outside the target

**Fig. 10:**
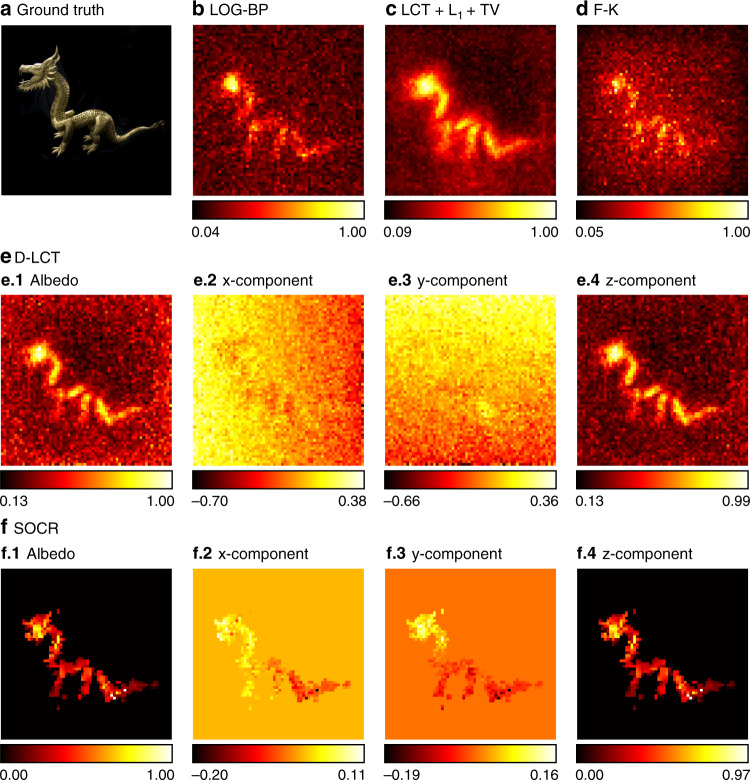
Reconstruction results of the dragon (confocal, 15 s). The ground truth is shown in **a**. Reconstructions are shown in **b**–**f**. Reconstruction results of the LOG-BP, F-K, and D-LCT methods are noisy. The LCT + L_1_ + TV reconstruction contains smooth background noise. The *x*-component and *y*-component of the D-LCT method are corrupted with noise. The proposed framework provides a faithful reconstruction of the target with little noise

In Figs. 9 and 10, we show reconstruction results of the instance of the dragon with a total exposure time of 60 min and 15 s, respectively. The specularity of the material and high-level noise in the measured data make it challenging to obtain fine reconstructions. For the case of a long exposure time, all methods find the target correctly. Our algorithm provides a clear reconstruction of the object with fine details and little noise due to the collaborative regularization. In extremely short exposure time, reconstructions of existing methods are of low quality and contain heavy noise, while the proposed method provides a faithful reconstruction of the hidden target. The head and tail of the dragon are well reconstructed with fine details.

**Fig. 11:**
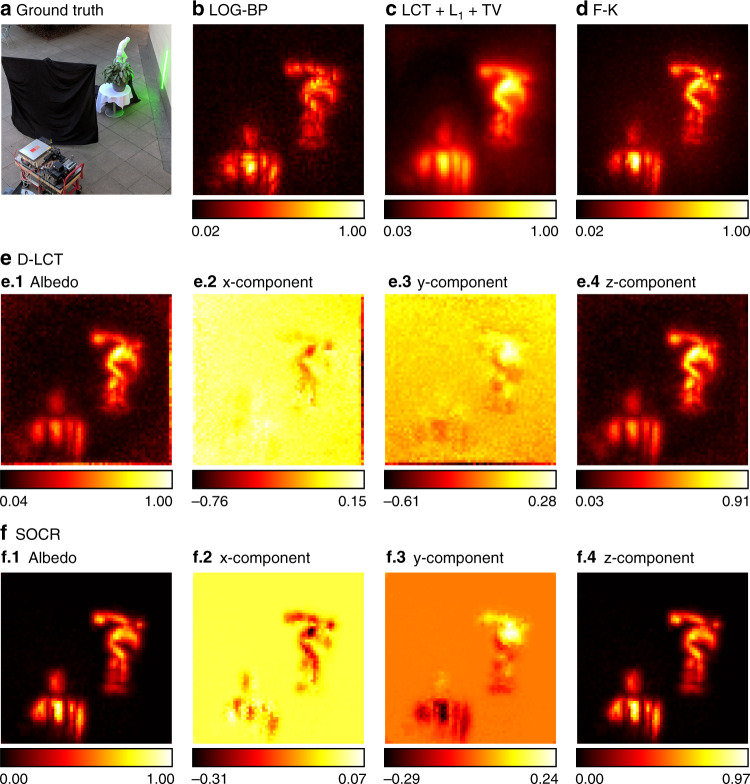
Reconstruction results of the outdoor scene (confocal, 10 min). The experimental set-up is shown in **a**. Reconstructions are shown in **b**–**f**. The LOG-BP reconstruction is of low quality. Both the LCT + L_1_ + TV and F-K reconstructions contain background noise. Our reconstruction has the highest contrast

To test the proposed framework for outdoor applications under confocal settings, we use the scenario containing a statue and a potted plant on the table in this dataset. The total exposure time is 10 min. As is shown in Fig. 11, the LOG-BP reconstruction is of low quality, with many discontinuous fragments. The LCT + L_1_ + TV and F-K reconstructions contain background noise. Both the D-LCT method and the proposed algorithm reconstruct the scene well, while our reconstruction is less noisy, especially in the *y*-component. Besides, we also provide a better reconstruction of the normal of the white tablecloth than the D-LCT method.

To test the proposed method on measured data under non-confocal settings, we use the instances of the NLOS letters, the shelf, and the office scene from the dataset provided by Liu et al.^8^. The time resolution is downsampled to 16 ps. We also convert the non-confocal measurements to confocal signals using the midpoint approximation technique to bring the LCT + L_1_ + TV, F-K, and D-LCT methods into comparison. For the instances of the NLOS letters and the shelf, the visible surface is illuminated at 130 × 180 points and the distance of the adjacent sampling grids is 0.01 m. The photons are detected at a fixed point, which is 1.05 m to the left and 0.73 m to the top of the sampling region. The exposure time is 390 min in total. For the instance of the office scene, the visible surface is illuminated at 131 × 181 points. The photons are detected at a fixed point, which is 1.04 m to the left and 0.61 m to the top of the sampling region. The exposure time per pixel measurement is 1 ms and it takes only 23 s for the whole measurements.

**Fig. 12:**
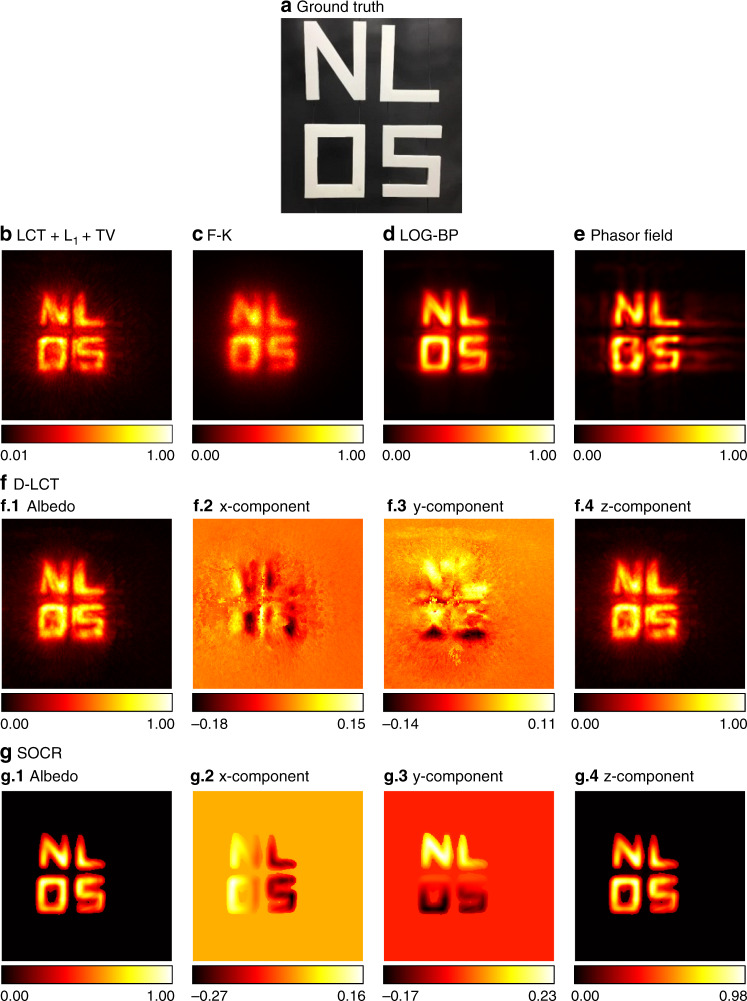
Reconstruction results of the NLOS letters (non-confocal, 390 min). The ground truth is shown in **a**. Reconstructions are shown in **b**–**g**. Reconstruction results of the LCT + L_1_ + TV, LOG-BP, phasor field and D-LCT methods contain artifacts. The SOCR reconstruction is noiseless, and the gaps between the four letters are clearly reconstructed

In Fig. 12 we compare our reconstruction result of the letters NLOS with existing reconstruction algorithms. The LOG-BP method provides a blurry reconstruction of the gap between the letters ‘N’ and ‘O’. The phasor field reconstruction is sharp, but with artifacts outside the ground truth. The F-K, LCT + L_1_ + TV, and D-LCT reconstructions are noisy and contain artifacts. This indicates the fact that the approximation error in the process of converting non-confocal measurements to confocal signals has considerable influence and cannot be neglected. Our reconstruction captures the four letters correctly and stands out as the only one that reconstructs the gaps between the four letters clearly.

**Fig. 13:**
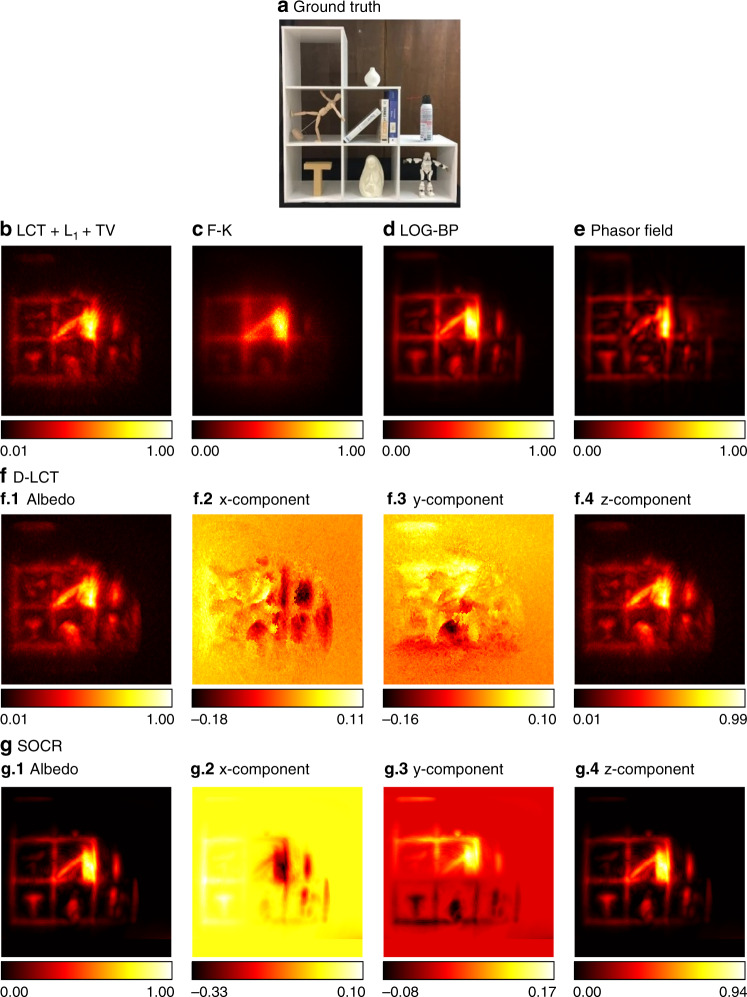
Reconstruction results of the shelf with lights on (non-confocal, 390 min). The ground truth is shown in **a**. Reconstructions are shown in **b**–**g**. The hidden scene is measured under strong ambient light. Both the LOG-BP method and the SOCR algorithm reconstruct the targets well, while SOCR also reconstructs the surface normal of the hidden scene

In Fig. 13 we show reconstruction results of the instance of the shelf, which is a complex scenario. The measurements are obtained with all the lights on^7^. The reconstruction results of the F-K, LCT + L_1_ + TV and D-LCT methods are blurry and noisy. This phenomenon can result from the approximation error in the process of converting the non-confocal measurements to confocal signals. The bottle in the phasor field reconstruction is over smoothed and the stone next to the letter T is not correctly reconstructed. Both the LOG-BP method and the SOCR algorithm reconstruct the targets well, while SOCR also reconstructs the surface normal of the hidden scene.

**Fig. 14:**
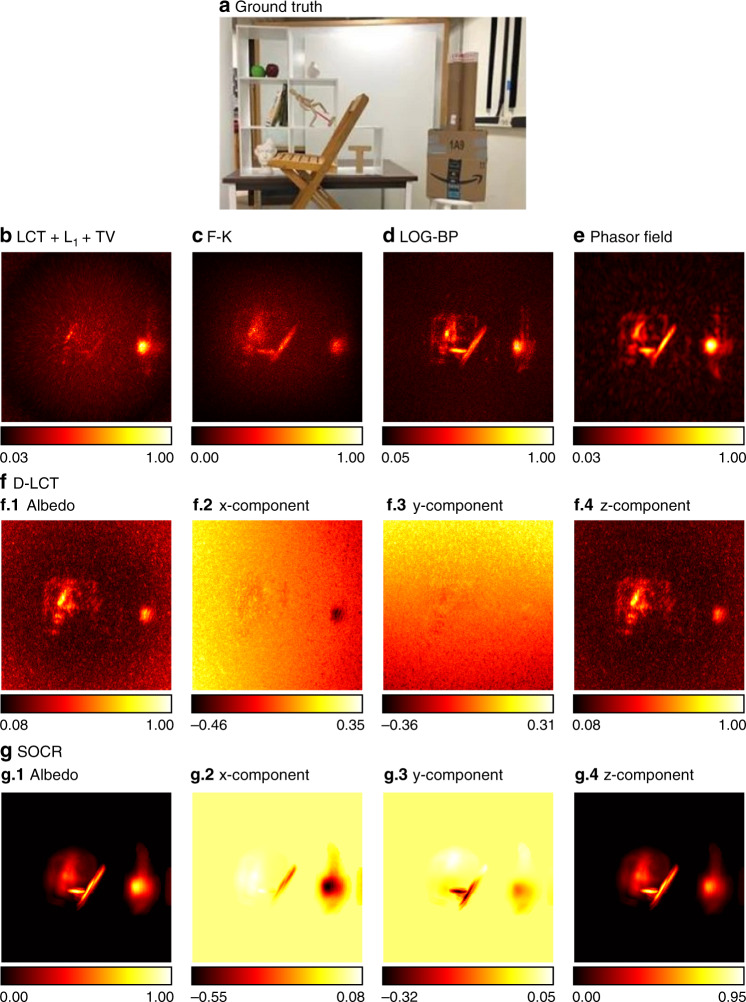
Reconstruction results of the office scene (non-confocal, 23 s). The ground truth is shown in **a**. Reconstructions are shown in **b**–**g**. In this scene, the shelf is partially occluded by the chair, which makes it hard to reconstruct fine details. Reconstructed targets obtained with the LCT + L_1_ + TV, F-K and D-LCT methods are noisy. The LOG-BP and phasor field reconstructions contain artifacts. The proposed method provides a smooth reconstruction of the scene

In Fig. 14 we compare reconstruction results of the instance of the office scene. The D-LCT method fails to reconstruct the chair correctly. The F-K and LCT + L_1_ + TV reconstructions are noisy. The LOG-BP and phasor field reconstruction contain artifacts in the background. The proposed framework provides a smooth reconstruction of the scene.

## Discussion

We have proposed a signal–object collaborative regularization based optimization framework that provides accurate estimations of both the albedo and surface normal under confocal and non-confocal NLOS settings. Reconstructions of the proposed method have sharp boundaries and contain very little noise.

### Compatibility with the physical model

In our framework, the reconstruction task is accomplished by solving an optimization problem with data fidelity and joint regularization. It can be used as a plug-in module in different physical models^25,29,31^. In addition, the proposed collaborative regularization term can be further simplified to accommodate cases where only the albedo needs to be reconstructed.

### Choice of the parameters

The proposed method involves several regularization parameters. To reduce the difficulty of solving the system, we decompose the optimization problem into sub-problems. Many of them are closely related to image denoising problems where the choice of the parameters is well studied^33,39^. Most of the parameters are determined adaptively and automatically. In Section 3 of the Supplement, we provide a detailed discussion of the choice of parameters.

### Complexity and execution time

In the proposed framework, the reconstruction is realized by solving an optimization problem with orthogonal constraints. This problem is solved using alternating iterations, which pays a high cost of increased computations. When the reconstruction domain is discretized by N × N × N voxels and the visible wall is sampled at N × N points, the time and memory complexity are O(N^5^) and O(N^3^), respectively. It takes about 3 min to reconstruct the instance of the letter K on an Intel Xeon Gold 5218 server with 64 cores. More details are provided in section 4 of the supplement. The proposed framework is easy to implement using embarrassingly parallel algorithms^40^. Compared to the reconstruction quality improved, the computational time could be regarded as secondary in importance, considering the growing computational capabilities and possible implementations on large-scale parallel computing platforms.

### Convergence analysis

The proposed constrained optimization problem (14) is highly nonlinear and nonconvex due to the L_1_ regularization term and the two orthogonal constraints. It is decomposed into sub-problems and solved approximately (see Section 2 of the Supplement). In the initializing stage, a convex least-squares problem is solved using the conjugate gradient method, so the final reconstruction is not sensitive to the initial value. In all experiments, we use zero values as an initialization of the hidden targets. Then, an L_1_ regularized problem is solved efficiently using the split Bregman method with convergence guarantee^41^. In the sub-problem of dictionary learning, we use the discrete cosine matrices as initial values of the two orthogonal dictionaries and update the dictionaries and the coefficients iteratively. The orthogonality constraints are preserved in each iteration and the corresponding objective value decreases monotonically^34^. The sub-problem of updating the estimated signal is also solved iteratively and the corresponding objective functions are convex. Convergence of the sub-problem of updating the reconstructed target is also guaranteed using the split Bregman method. The global convergence is not obtained, because the reconstructed target is updated approximately (see Section 2 of the Supplement). Nonetheless, numerical experiments indicate the empirical convergence of the proposed algorithm.

### Feature extraction of the reconstructed target

**Fig. 15:**
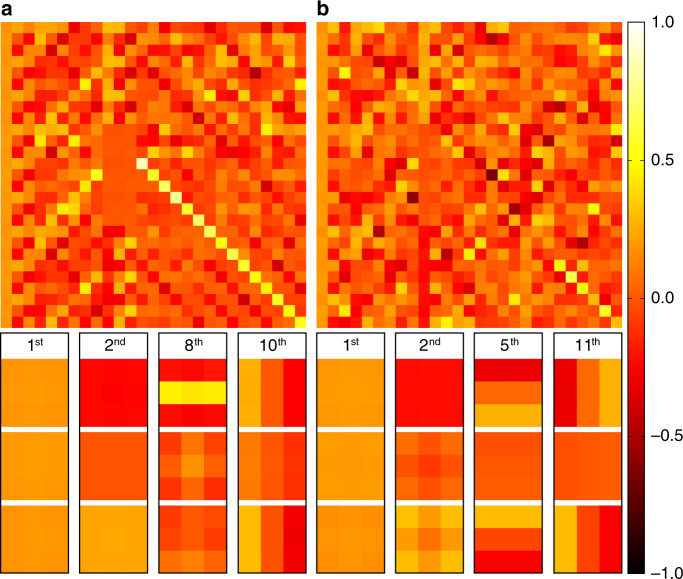
The spatial dictionaries learned from the instances of the letter T and the pyramid. The spatial dictionary learned from the instance of the letter T is shown in **a**. The spatial dictionary learned from the instance of the pyramid is shown in **b**. For the letter T, the first atom captures the low-frequency pattern of the local patches. The second, eighth, and tenth atoms capture the local structures of the object in three directions. For the instance of the pyramid, the dictionary atoms provide an accurate representation of the four faces. These two dictionaries differ significantly and can be used in further tasks like recognition and classification

In the proposed collaborative regularization term, two dictionaries are used to capture the local structures and non-local correlations of the reconstructed target. In Fig. 15 we show the spatial dictionaries learned from the instances of the letter T and the pyramid. The dictionary atoms are of size 3 × 3 × 3 and are shown in the vector form in each column of the matrices. For each instance, four atoms are shown in detail in the form of slices parallel to the visible wall. The atoms of the letter T capture the vertical and horizontal structures of the target, while the atoms learned from the instance of the pyramid capture the orientations of its four faces. The dictionary atoms and their corresponding coefficients can be viewed as features of the reconstructed target, which can be used for further tasks, such as recognition and classification.

### Necessity of introducing the joint prior

**Fig. 16:**
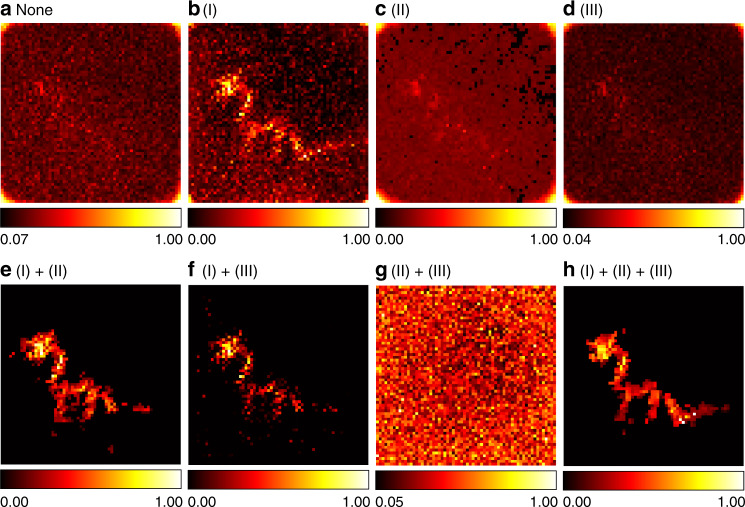
Reconstruction results of the dragon with different regularizations (confocal, 15 s). Reconstruction without regularization is shown in **a**. Reconstructions with different regularization settings are shown in **b**–**h**. The measurements contain heavy noise due to extreme short exposure time. The specularity of the material also makes the physical model deviate from the measurement process. The proposed joint signal–object prior is a combination of three priors, namely (I) the sparseness prior of the target, (II) the non-local self-similarity prior of the target, and (III) the smoothness prior of the signal

The proposed joint signal–object prior is a combination of three priors, namely (I) the sparseness prior of the target, (II) the non-local self-similarity prior of the target, and (III) the smoothness prior of the signal. To demonstrate the necessity of introducing them all, in Fig. 16 we show reconstruction results of the instance of the dragon in 15 s exposure time under different regularization settings. As is shown in Fig. 16a, when no prior is introduced, the solution of the least-squares problem is of low quality due to high measurement noise, and one can hardly identify the dragon from background noise. When the sparseness prior of the object is used, the visual quality is much better, but the reconstruction still contains background noise (Fig. 16b). As is shown in Fig. 16c and d, introducing the non-local self-similarity prior or the smoothness prior alone only brings minor improvements. In the absence of the smoothness prior or the non-local self-similarity prior, the reconstructions contain artifacts (Fig. 16e) or discontinuities (Fig. 16f). In the absence of the sparseness prior, the dictionary learning stage actually learns the background noise, and the reconstruction does not contain the hidden target (Fig. 16g). As is shown in Fig. 16h, a faithful reconstruction of the hidden target is obtained with collaborative regularization, even in the presence of high measurement noise.

## Materials and methods

The NLOS reconstruction process depends on the physical model used. Rather than putting forward a new physical model, we simplify the model introduced by Tsai et al. ^31^ as

1 $$\begin{array}{ll}\tau \left( {x^{\prime}_{\rm {i}},y^{\prime}_{\rm {i}},x^{\prime}_{\rm {d}},y^{\prime}_{\rm {d}},t} \right)\,=\,{\int}\!{\mathop {\iint}\nolimits_\Omega {\frac{{\left( {x^{\prime}_{\rm {i}} - x,y^{\prime}_{\rm {i}}-y,-z} \right) \cdot {\mathbf{n}}\left( {x,y,z,:}\right)}}{{d\left({x^{\prime}_{\rm {i}},y^{\prime}_{\rm {i}},x,y,z} \right)^3}}}}\\\qquad\qquad\qquad\qquad\quad\cdot \,\frac{{\left( {x^{\prime}_{\rm {d}}-x,y^{\prime}_{\rm {d}}-y,-z}\right)\cdot{\mathbf{n}}\left( {x,y,z,:}\right)}}{{d\left( {x^{\prime}{\rm{d}},y^{\prime}_{\rm {d}},x,y,z} \right)^3}}f\left( {x,y,z}\right)\\\qquad\qquad\qquad\qquad\quad\cdot\,\delta\left( {d\left( {x^{\prime}_{\rm {i}},y^{\prime}_{\rm {i}},x,y,z}\right)+d\left( {x^{\prime}_{\rm {d}},y^{\prime}_{\rm {d}},x,y,z}\right) -ct}\right){\rm {d}}x{\rm {d}}y{\rm {d}}z\end{array}$$

in which $c$ is the speed of light, the visible wall is positioned at the plane $z = 0$, $x^{\prime}_{\rm{i}}$ and $y^{\prime}_{\rm{i}}$ are the coordinates of the illumination point on the visible wall, $x^{\prime}_{\rm{d}}$ and $y^{\prime}_{\rm{d}}$ are the coordinates of the detection point on the visible wall. $\tau \left( {x^{\prime}_{\rm{i}},y^{\prime}_{\rm{i}},x^{\prime}_{\rm{d}},y^{\prime}_{\rm{d}},t} \right)$ is the photon intensity measured at time $t$. $f(x,y,z)$ is the albedo value at the point $(x,y,z)$, ${{{\mathbf{n}}}}(x,y,z,:)$ is a vector that represents the unit surface normal pointing toward the visible wall at the point $(x,y,z)$. $\delta$ represents the Dirac delta function. The distances between the point $(x,y,z)$ in the reconstructed domain and the illumination and detection points are given by

2 $$d\left( {x^{\prime}_{\rm{i}},y^{\prime}_{\rm{i}},x,y,z} \right) = \sqrt {\left( {x^{\prime}_{\rm{i}} - x} \right)^2 + \left( {y^{\prime}_{\rm{i}} - y} \right)^2 + z^2}$$

3 $$d\left( {x^{\prime}_{\rm {d}},y^{\prime}_{\rm {d}},x,y,z} \right) = \sqrt {\left( {x^{\prime}_{\rm {d}} - x} \right)^2 + \left( {y^{\prime}_{\rm {d}} - y} \right)^2 + z^2}$$

In Eq. (1), the measurement is nonlinear with respect to the surface normal. We simplify this model as

4 $$\begin{array}{ll}\tau_{\rm{s}}\left( {x^{\prime}_{\rm{i}},y^{\prime}_{\rm{i}},x^{\prime}_{\rm {d}},y^{\prime}_{\rm{d}},t} \right) ={\int}\!{\mathop{\iint}\nolimits_\Omega {\frac{{\left( {x^{\prime}_{\rm {d}} - x,y^{\prime}_{\rm{d}}-y,-z}\right)\cdot{\mathbf{n}}\left({x,y,z,:} \right)}}{{d\left({x^{\prime}_{\rm{i}},y^{\prime}_{\rm{i}},x,y,z}\right)^2 d\left( {x^{\prime}_{\rm {d}},y^{\prime}_{\rm {d}},x,y,z}\right)^3}}f\left( {x,y,z}\right)}}\\\qquad\qquad\qquad\qquad\quad \cdot\,\delta \left({d\left( {x^{\prime}_{\rm{i}},y^{\prime}_{\rm{i}},x,y,z}\right)+d\left({x^{\prime}_{\rm {d}},y^{\prime}_{\rm{d}},x,y,z} \right)-ct} \right){\rm {d}}x{\rm {d}}y{\rm {d}}z\end{array}$$

By denoting ${{{\mathbf{u}}}} = f{{{\mathbf{n}}}}$, Eq. (4) can be rewritten as

5 $$\begin{array}{ll}\tau_{\rm {s}}\left( {x^{\prime}_{\rm{i}},y^{\prime}_{\rm{i}},x^{\prime}_{\rm {d}},y^{\prime}_{\rm {d}},t} \right)\,={\int}\!{\mathop {\iint}\nolimits_\Omega {\frac{{\left( {x^{\prime}_{\rm{d}}-x,y^{\prime}_{\rm {d}}-y,-z}\right)\cdot{\mathbf{u}}\left( {x,y,z,:}\right)}}{{d\left( {x^{\prime}_{\rm{i}},y^{\prime}_{\rm{i}},x,y,z}\right)^2d\left( {x^{\prime}_{\rm {d}},y^{\prime}_{\rm {d}},x,y,z} \right)^3}}} }\\\qquad\qquad\qquad\qquad\quad\cdot \,\delta \left( {d\left({x^{\prime}_{\rm{i}},y^{\prime}_{\rm{i}},x,y,z}\right)+d\left( {x^{\prime}_{\rm{d}},y^{\prime}_{\rm {d}},x,y,z}\right)-ct}\right){\rm {d}}x{\rm{d}}y{\rm {d}}z\end{array}$$

which is a linear model with respect to the variable ${{{\mathbf{u}}}}$. For the special case of the confocal settings, we have $x^{\prime} = x^{\prime}_{\rm{i}} = x^{\prime}_{\rm{d}}$ and $y^{\prime} = y^{\prime}_{\rm{i}} = y^{\prime}_{\rm{d}}$. Equation (5) reduces to the directional-albedo model^24^

6 $$\begin{array}{ll}\tau_{\rm{{s,con}}}\left( {x^\prime,y^\prime,t}\right)\,={\int}\!{\mathop {\iint}\nolimits_\Omega{\frac{{\left({x^{\prime}-x,y^{\prime}-y,-z}\right)\cdot {{{\mathbf{u}}}}\left({x,y,z,:}\right)}}{{d\left({x^\prime,y^\prime ,x,y,z}\right)^5}}}}\\\qquad\qquad\qquad\qquad\quad\cdot\,\delta\left({2d\left({x^\prime,y^\prime,x,y,z}\right)-ct}\right){\rm{d}}x{\rm {d}}y{\rm{d}}z\end{array}$$

In both confocal and non-confocal cases, we find the vector field ${{{\mathbf{u}}}}$ that matches the corresponding measurements. Then, the albedo and surface normal of the reconstructed target can be computed as ${{{\mathbf{L}}}} = \left\| {{{\mathbf{u}}}} \right\|$ and ${{{\mathbf{n}}}} = \frac{{{{\mathbf{u}}}}}{{\left\| {{{\mathbf{u}}}} \right\|}}$. The surface normal is not defined where the albedo is zero.

The reconstruction of the vector field ${{{\mathbf{u}}}}$ can be obtained by solving the regularized least-squares problem

7 $${{{\mathbf{u}}}}^ \ast = \mathop {{{{{\mathrm{arg}}}}\min }}\limits_{{{\mathbf{u}}}} \;\left\| {A{{{\mathbf{u}}}} - {{{\tilde{\mathbf d}}}}} \right\|^2 + \Gamma \left( {{{\mathbf{u}}}} \right)$$

in which $A$ is the forward model described in Eq. (5) for the non-confocal settings or Eq. (6) for the confocal settings, ${\tilde{{\mathbf{d}}}}$ represents the raw measurement and $\Gamma ({{{\mathbf{u}}}})$ is a regularization term of the reconstruction ${{{\mathbf{u}}}}$.

In real-world applications, the measurements are corrupted with noise unavoidably, which may lead to noisy reconstructions. To tackle this problem, we introduce the estimated signal ${{{\mathbf{d}}}}$ as an approximation of the oracle signal corresponding to the real hidden scene and use the raw measurements as a source that provides partial information of the estimated signal. Joint priors for ${{{\mathbf{d}}}}$ and ${{{\mathbf{u}}}}$ are designed to obtain high-quality reconstructions. The proposed framework is given by

8 $$\left( {{{{\mathbf{u}}}}^ \ast ,{{{\mathbf{d}}}}^ \ast } \right) = \mathop {{{{{\mathrm{arg}}}}\min }}\limits_{{{{\mathbf{u}}}},{{{\mathbf{d}}}}} {\mkern 1mu} {\mkern 1mu} \left\| {A{{{\mathbf{u}}}} - {{{\mathbf{d}}}}} \right\|^2 + J\,\left( {{{{\mathbf{u}}}},{{{\mathbf{d}}}}} \right)$$

in which $J({{{\mathbf{u}}}},{{{\mathbf{d}}}})$ is the collaborative regularization term containing the raw measurement $\tilde{\mathbf{d}}$.

In this work, the joint prior we construct is based on three assumptions: sparseness of the hidden surface, self-similarity of the hidden object, and smoothness of the estimated signal. The regularization term is formulated as a weighted combination of three priors and the optimization problem is solved using the alternating iteration method.

The first prior focuses on the sparseness of the hidden scene. To recover the unseen objects, we use discrete voxels in three dimensions. However, it is only possible to reconstruct the surface of the hidden object where photons can reach. For this reason, the directional albedo is sparse. We impose the sparseness on the albedo and the first prior is

9 $$J_1({{{\mathbf{u}}}}) = \mathop {\sum}\limits_{i_1,i_2,i_3} {\sqrt {\mathop {\sum}\limits_{j = 1}^3 {{{\mathbf{u}}}} \left( {i_1,i_2,i_3,j} \right)^2} } = \mathop {\sum}\limits_{i_1,i_2,i_3} {{{{\mathbf{L}}}}\left( {i_1,i_2,i_3} \right)}$$

in which ${{{\mathbf{L}}}}$ represents the albedo. $i_1$, $i_2$ and $i_3$ are indices of the voxels in three directions.

The second prior is introduced to capture local structures of the hidden target. It is assumed that the hidden scene is subject to a non-local self-similarity prior, which means that local structures repeat many times in the reconstruction domain. To preserve the orientation of the surface, we impose this prior on the albedo ${{{\mathbf{L}}}}$. We call a sub-block matrix of the albedo ${{{\mathbf{L}}}}$ a local spatial patch. For each reference patch with the voxel $(i_1,i_2,i_3)$ in the left, top and front, we find its $H$ nearest neighbors in terms of root mean square error and call these patches the neighboring patches of the reference patch. Then, these patches are stretched into vectors and listed column by column to form a matrix such that their similarities with the reference patch are in descending order. We denote this matrix by $B_{i_1,i_2,i_3}({{{\mathbf{L}}}})$. Our goal is to find two orthogonal matrices that sparsely represent the local spatial structures (columns) and non-local correlations (rows) of the targets. This sparse approximation scheme can be intuitively written by

10 $$B_{i_1,i_2,i_3}\left( {{{\mathbf{L}}}} \right) \approx D_{\rm{s}}C_{i_1,i_2,i_3}D_{\rm{n}}^{ {T}}$$

in which $C_{i_1,i_2,i_3}$ is the sparse matrix that consists of transform coefficients, $D_{ \rm{s}}$ and $D_{\rm{n}}$ are orthogonal matrices. The second regularization term is given by

11 $$J_2\left({{{\mathbf{u}}}}\right)=\mathop{\sum}\limits_{i_1,i_2,i_3}{\left({\begin{array}{*{20}{c}}{\left\|{B_{i_1,i_2,i_3}\left({{{\mathbf{L}}}}\right)-D_{\rm{s}}C_{i_1,i_2,i_3}D_{\rm{n}}^{{T}}}\right\|^2 +\lambda _{{{pu}}}^2\left| {C_{i_1,i_2,i_3}}\right|_0}\end{array}}\right)}$$

in which the summation is over all possible blocks. $i_1$, $i_2$ and $i_3$ are indices of the voxel in the left, top and front of the reference patch, $\left| {C_{i_1,i_2,i_3}} \right|_0$ is the number of nonzero values of $C_{i_1,i_2,i_3}$ and $\lambda _{{ {pu}}}$ is a fixed parameter that controls sparsity of the transform coefficients.

The third prior concerns smoothness of the estimated signal. Since noisy data usually lead to noisy reconstruction, we introduce the variable ${{{\mathbf{d}}}}$ as an approximation of the ideal signal. We denote by $P_{(i_1,i_2,i_3)}({{\tilde{\mathbf{d}}}})$ the vector form of a patch of the raw measurement, which is a sub-block of the measured data. $(i_1,i_2,i_3)$ represents the indices of the voxel of this patch in the left, top and front. In the ideal Wiener filter, the penalty is imposed on coefficients in the frequency domain with weights determined by the oracle signal and the noise level. In real applications, the oracle signal is not known, but an approximation can be obtained with the reconstruction. Based on this observation, the third prior is given by

12 $$\begin{array}{l}J_3({{{\mathbf{u}}}},{{{\mathbf{d}}}}) = \left\| {{{{\mathbf{d}}}} - {{{\tilde{\mathbf d}}}}} \right\|^2 + \lambda _{{ {pd}}}\lambda _{{ {sd}}}\mathop {\sum}\limits_{i_1,i_2,i_3} {\left\| {P_{\left( {i_1,i_2,i_3} \right)}({{{\mathbf{d}}}}) - D{{{\mathbf{S}}}}_{(i_1,i_2,i_3)}} \right\|^2} \\ \qquad\begin{array}{*{20}{c}} {\begin{array}{*{20}{c}} {} & {} \end{array}} & {} \end{array} + \lambda _{{{pd}}}\mathop {\sum}\limits_{i_1,i_2,i_3} {\left\| {P_{\left( {i_1,i_2,i_3} \right)}({{{\tilde{\mathbf d}}}}) - D{{{\mathbf{S}}}}_{(i_1,i_2,i_3)}} \right\|^2} \\ \qquad\begin{array}{*{20}{c}} {\begin{array}{*{20}{c}} {} & {} \end{array}} & {} \end{array} + \lambda _{{{pd}}}\mathop {\sum}\limits_{i_1,i_2,i_3,j} {\left( {\frac{\sigma }{{d_j^{\rm {T}}P_{\left( {i_1,i_2,i_3} \right)}(A{{{\mathbf{u}}}})}}{{{\mathbf{S}}}}_{(i_1,i_2,i_3)}\left( j \right)} \right)^2} \end{array}$$

in which ${{\tilde{\mathbf{d}}}}$ stands for the noisy measurement, $i_1$, $i_2$ and $i_3$ are indices of the voxel in the left, front, and top of the patch. $P_{(i_1,i_2,i_3)}({{{\mathbf{d}}}})$, $P_{(i_1,i_2,i_3)}( {{{\tilde{\mathbf{d}}}}})$ and $P_{(i_1,i_2,i_3)}(A{{{\mathbf{u}}}})$ are patches of the estimated signal, raw measurement and the simulated data generated by the reconstruction with the physical model respectively. $D$ represents the Kronecker product of the discrete cosine transform matrices in three spatial directions with its *j*^th^ filter denoted by $d_j$. ${{{\mathbf{S}}}}_{(i_1,i_2,i_3)}$ is the vector consisting of the corresponding transform coefficients in the frequency domain with its *j*^th^ element denoted by ${{{\mathbf{S}}}}_{(i_1,i_2,i_3)}(j)$. $\lambda _{{{pd}}}$, $\lambda _{{{sd}}}$ and $\sigma$ are fixed parameters. The first two terms provide a balance between the noisy measurement and the signal estimated by the empirical Wiener filter. The last two terms correspond to Wiener filtering. In this formulation, a better approximation of the oracle signal can be obtained, which in turn helps to improve the quality of the reconstructed target.

Finally, we formulate the collaborative regularization term as a weighted combination of these three prior terms.

13 $$J({{{\mathbf{u}}}},{{{\mathbf{d}}}}) = s_{{u}}J_1({{{\mathbf{u}}}}) + \lambda _{ {u}}J_2({{{\mathbf{u}}}}) + \lambda _{{d}}J_3({{{\mathbf{u}}}},{{{\mathbf{d}}}})$$

in which $s_u$, $\lambda _{{u}}$ and $\lambda _{{d}}$ are fixed parameters. The proposed SOCR reconstruction model is then written as

14 $$\begin{array}{ll}\mathop {{\min }}\limits_{{{{\mathbf{u}}}},{{{\mathbf{d}}}},D_{\rm {s}},D_{\rm {n}},C,{{{\mathbf{S}}}}} \left\| {A{{{\mathbf{u}}}} - {{{\mathbf{d}}}}} \right\|^2 + s_{{u}}\mathop {\sum}\limits_{i_1,i_2,i_3} {{{{\mathbf{L}}}}\left( {i_1,i_2,i_3} \right)} \\ \begin{array}{*{20}{c}} {\begin{array}{*{20}{c}} {} & {} \end{array}} & {} \end{array} + \lambda _{{u}}\mathop {\sum}\limits_{i_1,i_2,i_3} {\left( {\begin{array}{*{20}{c}} {\left\| {B_{i_1,i_2,i_3}\left( {{{\mathbf{L}}}} \right) - D_{\rm {s}}C_{i_1,i_2,i_3}D_{\rm {n}}^{\rm {T}}} \right\|^2 + \lambda _{{ {pu}}}^2\left| {C_{i_1,i_2,i_3}} \right|_0} \end{array}} \right)} \\ \begin{array}{*{20}{c}} {\begin{array}{*{20}{c}} {} & {} \end{array}} & {} \end{array} + \lambda _{ {d}}\left\| {{{{\mathbf{d}}}} - {{{\tilde{\mathbf d}}}}} \right\|^2 + \lambda _{ {d}}\lambda _{ {{pd}}}\mathop {\sum}\limits_{i_1,i_2,i_3} {\left\| {P_{\left( {i_1,i_2,i_3} \right)}({{{\tilde{\mathbf d}}}}) - D{{{\mathbf{S}}}}_{(i_1,i_2,i_3)}} \right\|^2} \\ \begin{array}{*{20}{c}} {\begin{array}{*{20}{c}} {} & {} \end{array}} & {} \end{array} + \lambda _{ {d}}\lambda _{{ {pd}}}\mathop {\sum}\limits_{i_1,i_2,i_3,j} {\left( {\frac{\sigma }{{d_j^{\rm {T}}P_{\left( {i_1,i_2,i_3} \right)}(A{{{\mathbf{u}}}})}}{{{\mathbf{S}}}}_{(i_1,i_2,i_3)}\left( j \right)} \right)^2} \\ \begin{array}{*{20}{c}} {\begin{array}{*{20}{c}} {} & {} \end{array}} & {} \end{array} + \lambda _{ {d}}\lambda _{{ {pd}}}\lambda _{{ {sd}}}\mathop {\sum}\limits_{i_1,i_2,i_3} {\left\| {P_{\left( {i_1,i_2,i_3} \right)}({{{\mathbf{d}}}}) - D{{{\mathbf{S}}}}_{(i_1,i_2,i_3)}} \right\|^2} \\ {\rm {s.t.}}\quad D_{\rm {n}}^{\rm {T}}D_{\rm {n}} = I_{p_xp_yp_z}\quad D_{\rm {s}}^{\rm {T}}D_{\rm {s}} = I_H{\mkern 1mu} \\ \begin{array}{*{20}{c}} {} & {} \end{array}{\mkern 1mu} {{{\mathbf{L}}}}\left( {i_1,i_2,i_3} \right) = \sqrt {\mathop {\sum}\limits_{j = 1}^3 {{{\mathbf{u}}}} \left( {i_1,i_2,i_3,j} \right)^2} \end{array}$$

in which $p_x$, $p_y$ and $p_z$ are sizes of the patches of the reconstructed albedo in three directions. $H$ is the number of neighbors selected for each patch. This problem is solved using the alternative iteration method with two stages, and the main steps are shown in Fig. 1c. In the initializing stage, a basic reconstruction is obtained by solving the least-squares problem. Then, the sparseness parameter is adaptively chosen and a sparse reconstruction is obtained by solving an L_1_-regularized problem. This reconstruction is used to initialize the dictionaries. In the second stage, the estimated signal, reconstructed target and dictionaries are updated iteratively to obtain the final reconstruction. In Section 2 of the Supplement, we provide a detailed discussion of the scheme to solve this problem.

## Supplementary information

Supplement

Dataset

## Data Availability

The Zaragoza dataset is available in *Zaragoza NLOS synthetic dataset* [http://graphics.unizar.es/nlos_dataset.html]. The Stanford dataset can be downloaded at the project page [http://www.computationalimaging.org/publications/nlos-fk/]. The NLoS benchmark dataset can be downloaded at the website [https://nlos.cs.uni-bonn.de/challenges/Geometry]. The dataset provided by the phasor field method is available at the project page [https://biostat.wisc.edu/~compoptics/phasornlos20/fastnlos.html].

## **Code availability**

Availability of the code is provided in the supplementary materials. The synthetic data of the instance of the pyramid are contained in our supplementary materials.
