# Supplementary material for: Non-line-of-sight reconstruction with signal–object collaborative regularization
Source: Light Sci Appl. 2021 Sep 24;10:198. doi: 10.1038/s41377-021-00633-3 (PMC8463571; doi:10.1038/s41377-021-00633-3)
Supplement: Supplementary file 1 — Supplement [file 41377_2021_633_MOESM1_ESM.pdf]

Supplementary Information for Non-Line-of-Sight Reconstruction with Signal-object Collaborative Regularization

Authors

Xintong Liu[1, †], Jianyu Wang[1, †], Zhupeng Li[2,3], Zuoqiang Shi[4,5], Xing Fu[2,3,*], Lingyun Qiu[1,5,*]

Affiliations

[1] Yau Mathematical Sciences Center, Tsinghua University, Beijing, China, 100084

[2] State Key Laboratory of Precision Measurement Technology and Instruments, Department of Precision Instrument, Tsinghua University, Beijing, China, 100084

[3] Key Laboratory of Photonic Control Technology (Tsinghua University), Ministry of Education, Beijing, China, 100084

[4] Department of Mathematical Sciences, Tsinghua University, Beijing, China, 100084

[5] Yanqi Lake Beijing Institute of Mathematical Sciences and Applications, Beijing, China, 101408

[†] These authors contribute equally to this work.

[*] Correspondence and requests for materials should be addressed to fuxing@tsinghua.edu.cn (Xing Fu) and lyqiu@tsinghua.edu.cn (Lingyun Qiu).

This supplement consists of four sections. In section 1, we provide additional reconstruction results. In section 2, we explain the SOCR algorithm in detail. In section 3, we show how the parameters are adaptively chosen. In section 4, we show the time and memory complexity of the proposed framework and compare the execution time. In all comparisons, we abbreviate the proposed signal-object collaborative regularization algorithm to SOCR.

1. Additional results

1.1 Signal preprocessing

Measurement noise is one of the major obstacles to obtain high-quality reconstructions. For the instance of the dragon with a total exposure time of 15 s from the Stanford dataset[1], we downsample the original measurement to 64×64 illumination points. We use the BM3D algorithm[2] to preprocess the measured data. Reconstruction results of different NLOS reconstruction algorithms using the denoised data are shown in Fig. S1. The LCT+L_1+TV[3] reconstruction contains heavy background noise. The FK[1], LOG-BP[4], and D-LCT[5] reconstructions are blurry. The proposed SOCR algorithm reconstructs the dragon with the highest contrast. All methods fail to reconstruct the boundary of the target precisely due to the bias introduced in the signal preprocessing step (See Fig. 10 in the article for reconstruction results without signal preprocessing).

1.2 Reconstructions of simulated data with noise

We use the instance of the letter T from the Zaragoza dataset[6] to test the robustness of the proposed SOCR method. The noisy signals are simulated using the Poisson model introduced in the LCT+L_1+TV method[3]. The maximum intensity of the noiseless signal is normalized to 100 and the levels of background noise are set from 0 to 1 000. For each noise level, 10 different signals are simulated and the average albedo RMSE are compared in Fig. S2. When the noise level is 1000 (ten times the maximum intensity of the noiseless signal), the mean albedo RMSE of the proposed method is 0.2055, which is smaller than that of the D-LCT method at noise level 300 (0.2183). Reconstruction results of the least-squares method without regularization, the SOCR method, and the D-LCT method are compared in Fig. S3.

2. The SOCR algorithm

The signal-object collaborative regularized NLOS reconstruction is obtained by solving the constrained least-squares problem.

$$
\begin{aligned}
&\min_{\mathbf{u},\mathbf{d},D_s,D_n,C,\mathbf{S}} \|A\mathbf{u}-\mathbf{d}\|^2 + s_u \sum_{i_1,i_2,i_3} \mathbf{L}(i_1,i_2,i_3) \\
&+\lambda_u \sum_{i_1,i_2,i_3} \left(\|B_{i_1,i_2,i_3}(\mathbf{L}) - D_s C_{i_1,i_2,i_3} D_n^T\|^2 + \lambda_{pu}^2 |C_{i_1,i_2,i_3}|_0 \right) \\
&+\lambda_d \left\|\mathbf{d}-\tilde{\mathbf{d}}\right\|^2 + \lambda_d \lambda_{pd} \sum_{i_1,i_2,i_3} \left\| P_{(i_1,i_2,i_3)}(\tilde{\mathbf{d}}) - D\mathbf{S}_{(i_1,i_2,i_3)} \right\|^2 \\
&+\lambda_d \lambda_{pd} \sum_{i_1,i_2,i_3,j} \left(\frac{\sigma}{d_j^T P_{(i_1,i_2,i_3)}(A\mathbf{u})} \mathbf{S}_{(i_1,i_2,i_3)}(j) \right)^2 \\
&+\lambda_d \lambda_{pd} \lambda_{sd} \sum_{i_1,i_2,i_3} \|P_{(i_1,i_2,i_3)}(\mathbf{d}) - D\mathbf{S}_{(i_1,i_2,i_3)}\|^2 \\
s.t.\quad & D_n^T D_n = I_{p_x p_y p_z} \quad D_s^T D_s = I_H \\
& \mathbf{L}(i_1,i_2,i_3) = \sqrt{\sum_{j=1}^{3} \mathbf{u}(i_1,i_2,i_3,j)^2}
\end{aligned}
\tag{S.1}
$$

in which $\mathbf{u}$ represents the reconstructed target, $\mathbf{d}$ is the estimated signal. s_u, λ_u, λ_{pu}, λ_d, λ_{pd}, λ_{sd} and σ are fixed parameters. The iterative scheme for solving this optimization problem is shown in Alg. 1. Here we discuss in detail how the sub-problems are solved.

2.1 Initializing the reconstructed target

In step (1.2), a sparse reconstruction is obtained by solving

$$\mathbf{u}^0 = \underset{\mathbf{u}}{\operatorname{argmin}} \| A\,\mathbf{u} - \mathbf{d}^0 \|^2 + s_u \sum_{i_1,i_2,i_3} \sqrt{\sum_{j=1}^{3} \mathbf{u}\,(i_1,i_2,i_3,j)^2} \tag{S.2}$$

The split Bregman method[7] can be used to solve this problem and the scheme is summarized in Alg. 2. To update $\mathbf{u}$, the conjugate gradient method is used to solve the least-squares problem. The variable $\mathbf{v}$ is updated with pointwise hard-thresholding.

2.2 Initializing the dictionaries

In step (1.4), we initialize the dictionaries that sparsely represent the local structures and nonlocal correlations of the albedo by solving the optimization problem with orthogonal constraints.

$$\begin{gathered}(D_s^0, D_n^0, C^0) = \underset{D_s, D_n, C}{\operatorname{argmin}} \sum_{i_1,i_2,i_3} \left(\| B_{i_1,i_2,i_3}(\mathbf{L}^0) - D_s C_{i_1,i_2,i_3} D_n^T \|^2 + \lambda_{pu}^2 |C_{i_1,i_2,i_3}|_0 \right) \\ s.t. \quad D_n^T D_n = I_{p_x p_y p_z} \quad D_s^T D_s = I_H \end{gathered} \tag{S.3}$$

To simplify the notation, we denote by $B = [B_{1,1,1}(\mathbf{L}^0), \ldots, B_{I_1,I_2,I_3}(\mathbf{L}^0)]$ the matrix consisting of the blocks of $\mathbf{L}$ and by C the corresponding coefficient matrix. I_1, I_2 and I_3 are the number of voxels in each direction. It suffices to solve the problem of the following type

$$\begin{gathered}(L^*, R^*, C^*) = \underset{L,R,C}{\operatorname{argmin}} \| B - LCR^T \|^2 + \lambda_{pu}^2 |C|_0 \\ s.t. \quad L^T L = I_{p_x p_y p_z} \quad R^T R = I_H \end{gathered} \tag{S.4}$$

which can be solved by generalizing the algorithm introduced by Cai et al.[8]

2.3 Updating the estimated signal

In step (2.1), we update the estimated signal by solving the optimization problem

$$\begin{aligned}(\mathbf{d}^{k+1}, \mathbf{S}^{k+1}) = \underset{\mathbf{d},\mathbf{S}}{\operatorname{argmin}} & \| A\,\mathbf{u}^k - \mathbf{d} \|^2 + \lambda_d \left\| \mathbf{d} - \tilde{\mathbf{d}} \right\|^2 \\ & + \lambda_d \lambda_{pd} \sum_{i_1,i_2,i_3} \left\| P_{(i_1,i_2,i_3)}(\tilde{\mathbf{d}}) - D\,\mathbf{S}_{(i_1,i_2,i_3)} \right\|^2 \\ & + \lambda_d \lambda_{pd} \lambda_{sd} \sum_{i_1,i_2,i_3} \| P_{(i_1,i_2,i_3)}(\mathbf{d}) - D\,\mathbf{S}_{(i_1,i_2,i_3)} \|^2 \\ & + \lambda_d \lambda_{pd} \sum_{i_1,i_2,i_3,j} \left(\frac{\sigma}{d_j^T P_{(i_1,i_2,i_3)}(A\,\mathbf{u}^k)} \mathbf{S}_{(i_1,i_2,i_3)}(j) \right)^2 \end{aligned} \tag{S.5}$$

in which $D = dct(p_z) \otimes dct(p_y) \otimes dct(p_x)$ is the Kronecker product of the discrete cosine matrices. p_x, p_y and p_z are sizes of the patch in three directions. To simplify

the notation, we denote by P^* the operator that aggregates the patches back to the signal with the value of each voxel estimated by averaging all its appearances in the block dataset. In this way, the term $\sum_{i_1,i_2,i_3}\|P_{(i_1,i_2,i_3)}(\mathbf{d}) - D\mathbf{S}_{(i_1,i_2,i_3)}\|^2$ can be replaced by $\|\mathbf{d} - P^*(DS)\|^2$ with a scaling parameter, where $\mathbf{S} = [\mathbf{S}_{(1,1,1)},\ldots,\mathbf{S}_{(I_1,I_2,I_3)}]$ is the matrix consisting of the transform coefficients. We summarize the scheme for solving this sub-problem in Alg. 3.

2.4 Updating the reconstructed target

In step (2.2), we update the reconstruction $\mathbf{u}$ by solving

$$
\begin{aligned}
(\mathbf{u}^{k+1},\mathbf{L}^{k+1}) = \underset{\mathbf{u}}{\operatorname{argmin}} \|A\mathbf{u} - \mathbf{d}^{k+1}\|^2 + s_u \sum_{i_1,i_2,i_3} \sqrt{\sum_{j=1}^{3} \mathbf{u}(i_1,i_2,i_3,j)^2} \\
+ \lambda_u \sum_{i_1,i_2,i_3} \|B_{i_1,i_2,i_3}(\mathbf{L}) - D_s^k C_{i_1,i_2,i_3}^k (D_n^k)^T\|^2 \\
+ \lambda_d \lambda_{pd} \sum_{i_1,i_2,i_3,j} \left(\frac{\sigma}{d_j^T P_{(i_1,i_2,i_3)}(A\mathbf{u})} \mathbf{S}_{(i_1,i_2,i_3)}^{k+1}(j) \right)^2 \\
s.t.\ \mathbf{L}(i_1,i_2,i_3) = \sqrt{\textstyle\sum_{j=1}^{3} \mathbf{u}(i_1,i_2,i_3,j)^2}
\end{aligned} \tag{S.6}
$$

This sub-problem is non-linear with respect to $\mathbf{u}$ and is solved approximately without considering the last term. Similar to the operator P^*, we denote by Q^* the operator that aggregates the blocks of $\mathbf{L}$ back to the reconstructed albedo. In this way, the term $\sum_{i_1,i_2,i_3}\|B_{i_1,i_2,i_3}(\mathbf{L}) - D_s^k C_{i_1,i_2,i_3}^k (D_n^k)^T\|^2$ can be replaced by $\|\mathbf{L} - Q^*(B_{sr}^k)\|^2$ with a scaling parameter, where $B_{sr}^k = [D_s^k C_{1,1,1}^k (D_n^k)^T, \ldots, D_s^k C_{I_1,I_2,I_3}^k (D_n^k)^T]$ is the matrix consisting of sparse representations of dictionary atoms. To simplify the notation, we set $\mathbf{L}_{enhanced}^k = Q^*(B_{sr}^k)$. The optimization problem (S.6) without the last term can be written equivalently as

$$
\begin{aligned}
(\mathbf{u}^{k+1},\mathbf{L}^{k+1}) = \underset{\mathbf{u},\mathbf{L}}{\operatorname{argmin}} \|A\mathbf{u} - \mathbf{d}^{k+1}\|^2 + s_u \sum_{i_1,i_2,i_3} \mathbf{L}(i_1,i_2,i_3) \\
+ \lambda_u \|\mathbf{L} - \mathbf{L}_{enhanced}^k\|^2 \\
s.t.\ \mathbf{L}(i_1,i_2,i_3) = \sqrt{\textstyle\sum_{j=1}^{3} \mathbf{u}(i_1,i_2,i_3,j)^2}
\end{aligned} \tag{S.7}
$$

To further simplify this problem, we denote by $\mathbf{u}_{enhanced}^k$ the vector field defined as

$$
\mathbf{u}_{enhanced}^k(i_1,i_2,i_3,:) = \mathbf{L}_{enhanced}^k(i_1,i_2,i_3) \frac{\mathbf{u}^k(i_1,i_2,i_3)}{\|\mathbf{u}^k(i_1,i_2,i_3,:)\|} \tag{S.8}
$$

and solve

$$
\begin{aligned}
\mathbf{u}^{k+1} = \underset{\mathbf{u}}{\operatorname{argmin}} \|A\mathbf{u} - \mathbf{d}^{k+1}\|^2 + s_u \sum_{i_1,i_2,i_3} \sqrt{\textstyle\sum_{j=1}^{3} \mathbf{u}(i_1,i_2,i_3,j)} \\
+ \lambda_u \|\mathbf{u} - \mathbf{u}_{enhanced}^k\|^2
\end{aligned} \tag{S.9}
$$

The solution to this sub-problem is given in Alg. 4.

3. Choice of the parameters

The seven parameters used in our collaborative regularization model are s_u, λ_u, λ_{pu}, λ_d, λ_{pd}, λ_{sd} and σ. Tuning these parameters by hand can be a very tedious task. However, these parameters can be set adaptively based on the raw measurement.

In step (1.2), the parameters s_u and μ are chosen adaptively. s_u is the parameter that controls the sparseness of the reconstructed target and is set to be

$$s_u = k_{sparse} \frac{\| A\mathbf{u}_0^0 - \mathbf{d}^0 \|^2}{\sum_{i_1,i_2,i_3} \mathbf{L}_0^0(i_1,i_2,i_3)} \tag{S.10}$$

in which k_{sparse} is an implicit parameter that balances the data fidelity term and the sparsity regularization term. Note that in Alg. 2 the vector $\mathbf{u}_0^0(i_1,i_2,i_3,:)$ is thresholded to zero when $\mu \geq \frac{s_u}{2\|\mathbf{u}_0^0(i_1,i_2,i_3,:)\|}$, we choose

$$\mu = \frac{s_u}{2I_1I_2I_3} \sum_{i_1,i_2,i_3} \frac{1}{\|\mathbf{u}_0^0(i_1,i_2,i_3,:)\|} \tag{S.11}$$

In step (1.4), we implicitly determine the parameter λ_{pu}. This parameter controls the sparseness of the dictionary coefficients. We compute the amplification factor as $k_{amplify} = \frac{255}{\max_{\{(i_1,i_2,i_3)\}}\{\mathbf{L}_0^0(i_1,i_2,i_3)\}}$ and map $\mathbf{L}_0^0$ linearly into the range $[0,255]$. The sub-problem (S.3) is closely related to sparse coding schemes used in image denoising tasks where the choice of parameters has been fully studied[2]. In most of the experiments, we choose $\epsilon_{object} = 40$ as the latent noise level and set $\lambda_{pu} = 2.6\epsilon_{object}$, as was suggested in previous works[2,8]. In cases of high measurement noise, one may increase the parameter ϵ_{object} to better attenuate the noise.

In step (2.1), we update the estimated signal and determine the parameters λ_d, λ_{pd} and λ_{sd} adaptively. The raw measurement $\tilde{\mathbf{d}}$ is mapped linearly into the range $[0,255]$ in advance. Note that the process of updating the coefficients $\mathbf{S}_{(i_1,i_2,i_3)}$ can be viewed as the application of an empirical Wiener filter, with the noisy signal $\frac{1}{1+\lambda_{sd}}\tilde{\mathbf{d}} + \frac{\lambda_{sd}}{1+\lambda_{sd}}\mathbf{d}_j^{k+1}$, the pilot estimation $A\mathbf{u}^k$, and the noise level $\frac{\sigma}{\sqrt{1+\lambda_{sd}}}$. To avoid large bias, we set $\lambda_{sd} = 0.25$. The signal $\mathbf{d}_{j+1}^{k+1}$ is updated as a convex combination of $A\mathbf{u}^k$, $\tilde{\mathbf{d}}$ and $P^*(DS)$. Now that the noise contained in $P^*(DS)$ is significantly lower than the raw measurement due to Wiener filtering, we choose the weights in the combination as $1:1:8$, and the corresponding parameters are $\lambda_d = 1$ and $\lambda_{pd} = 16$.

In step (2.2), we determine the parameter λ_u adaptively as

$$\lambda_u = k_{target}\frac{\| A\,\mathbf{u}_0^0 - \mathbf{d}^0 \|^2}{\| \mathbf{u}^0 - \mathbf{u}_{enhanced}^0 \|^2} \tag{S.12}$$

in which k_{target} is a latent parameter. In most of the experiments, we set $k_{target} = 1$.

4. Complexity and execution time

When the reconstruction domain is discretized with $N \times N \times N$ voxels and the visible wall is sampled at $N \times N$ points, the time and memory complexity of the SOCR algorithm are $O(N^5)$ and $O(N^3)$ respectively. Here we discuss the time complexity in detail.

In step (1.2), the least-squares problem is solved using the conjugate gradient method, with a complexity of $O(N^5)$. The hard-thresholding operations take $O(N^3)$. Thus, the overall complexity is $O(N^5)$.

In step (1.3), $O(N^3)$ is required to compute the albedo values.

In step (1.4), for each reference patch, the neighboring patches are searched in a window of size $W \times W \times W$. All the patches in this window are sorted based on their similarity with the reference patch and the Q nearest neighbors are used to form the block matrix. It takes $O(N^3(P^3+3\log W)W^3)$ to complete the patch stacking step, in which P is the patch size in three directions. With fixed dictionaries, it takes $O(N^3P^3Q(P^3+Q))$ to compute the dictionary coefficients. Updating the dictionaries takes $O(N^3QP^6)+O(N^3P^3Q^2)+O(P^9)+O(Q^3)$. When $P<N^{1/4}$, $Q<N^{1/4}$, $W<N^{1/3}$, the overall complexity is no more than $O(N^5)$.

In step (2.1), generating the signal with the current reconstruction takes $O(N^5)$. The Wiener filtering step takes $O(N^3S^6)$, in which S is the patch size of the transient image in three directions. The aggregation step takes $O(N^3S^3)$. The time complexity is $O(N^5) + O(N^3S^6)$ in total. When $S < N^{1/3}$, the overall complexity is $O(N^5)$.

In step (2.2), in order to compute $\mathbf{u}_{enhanced}$, the time complexity of transform-domain hard-thresholding and aggregation are $O(N^3P^3Q(P^3+Q))$ and $O(N^3P^3Q)$ respectively. The time complexity of updating $\mathbf{u}$ and $\mathbf{v}$ are $O(N^5)$ and $O(N^3)$ respectively. When $P<N^{1/4}$, $Q<N^{1/4}$, the overall complexity is $O(N^5)$.

In step (2.3), the time complexity of updating the dictionaries and coefficients is the same as step (1.4), which is no more than $O(N^5)$.

We conclude that the time complexity of the proposed framework is $O(N^5)$ when $P<N^{1/4}$, $Q<N^{1/4}$, $W<N^{1/3}$ and $S < N^{1/3}$.

In Table S1 we compare the computation time of different methods for the instance of the letter K (Fig. 7 in the article). The size of the transient image is $64\times64\times1024$. The code of all methods was run on an Intel Xeon Gold 5218 server with 64 cores. Computation time of the proposed SOCR algorithm is shown in Table S2. The number of iterations is set to be 3. After step (1.2), a sparse reconstruction is obtained. The computation complexity of this step is $O(N^5)$, which is the same as the overall SOCR algorithm.

Table S1 Comparisons of computation time for the instance of the letter K

Methods	Size of discrete voxels	Run time (s)
LOG-BP[4]	64×64×12	9
LCT+L_1+TV[3]	64×64×1024	188 (40 iterations)
F-K[1]	64×64×1024	2
D-LCT[5]	64×64×1024×3	11
Phasor Field[9]	64×64×10	6
SOCR	64×64×21×3	272 (3 iterations)

Table S2 Execution time of the SOCR algorithm for the instance of the letter K

Step	Algorithm	Complexity	Execution time (s)		
Stage 1: Initializing					
Step (1.1)	-	$O(N^3)$	< 1		
Step (1.2)	Alg. 2	$O(N^5)$	144		
Step (1.3)	-	$O(N^3)$	< 1		
Step (1.4)	-	$\leqslant O(N^5)$	5		
Stage 2: Alternating iterations			Iter 1	Iter 2	Iter 3
Step (2.1)	Alg. 3	$O(N^5)$	9	9	9
Step (2.2)	Alg. 4	$O(N^5)$	30	27	28
Step (2.3)	-	$\leqslant O(N^5)$	4	3	4

Reference

1. Lindell, D. B., Wetzstein, G. & O'Toole, M. Wave-based non-line-of-sight imaging using fast *f-k* migration. *ACM Trans. Graph.* **38**, 1–13 (2019).
2. Dabov, K., Foi, A., Katkovnik, V. & Egiazarian, K. Image denoising with block-matching and 3D filtering. in *Proceedings of SPIE - The International Society for Optical Engineering* 354–365 (2006).
3. O'Toole, M., Lindell, D. B. & Wetzstein, G. Confocal non-line-of-sight imaging based on the light-cone transform. *Nature* **555**, 338–341 (2018).
4. Laurenzis, M. & Velten, A. Feature selection and back-projection algorithms for nonline-of-sight laser–gated viewing. *J. Electron. Imaging* **23**, 1–6 (2014).
5. Young, S. I., Lindell, D. B., Girod, B., Taubman, D. & Wetzstein, G. Non-Line-of-Sight Surface Reconstruction Using the Directional Light-Cone Transform. in *2020 IEEE/CVF Conference on Computer Vision and Pattern Recognition (CVPR)* 1404–1413 (IEEE, 2020). doi:10.1109/CVPR42600.2020.00148.
6. Galindo, M., Marco, J., O'Toole, M., Wetzstein, G. & Jarabo, A. A dataset for benchmarking time-resolved non-line-of-sight imaging. Proceedings of the ACM SIGGRAPH 2019. Los Angeles California: ACM, 2019.
7. Goldstein, T. & Osher, S. The Split Bregman Method for L1-Regularized Problems. *SIAM J. Imaging Sci.* **2**, 323–343 (2009).
8. Cai, J. F., Ji, H., Shen, Z. & Ye, G. B. Data-driven tight frame construction and image denoising. *Appl. Comput. Harmon. Anal.* **37**, 89–105 (2014).
9. Liu, X., Bauer, S. & Velten, A. Phasor field diffraction based reconstruction for fast non-line-of-sight imaging systems. *Nat. Commun.* **11**, 1645 (2020).

Algorithm 1: The SOCR algorithm

Stage 1: Initialization

(1.1) Initializing the signal: $\mathbf{d}^0 = \tilde{\mathbf{d}}$

(1.2) Initializing the reconstruction:

$$\mathbf{u}^0 = \underset{\mathbf{u}}{\operatorname{argmin}} \|A\mathbf{u} - \mathbf{d}^0\|^2 + s_u \sum_{i_1,i_2,i_3} \sqrt{\sum_{j=1}^{3} \mathbf{u}(i_1,i_2,i_3,j)^2}$$

(1.3) Computing the albedo: $\mathbf{L}^0(i_1,i_2,i_3) = \sqrt{\sum_{j=1}^{3} \mathbf{u}^0(i_1,i_2,i_3,j)^2}$

(1.4) Initializing the dictionaries with $\mathbf{L}^0$:

$$(D_s^0, D_n^0, C^0) = \underset{D_s,D_n,C}{\operatorname{argmin}} \sum_{i_1,i_2,i_3} \left(\|B_{i_1,i_2,i_3}(\mathbf{L}^0) - D_s C_{i_1,i_2,i_3} D_n^T\|^2 + \lambda_{pu}^2 |C_{i_1,i_2,i_3}|_0 \right)$$

$$s.t. \quad D_s^T D_s = I_{p_x p_y p_z} \quad D_n^T D_n = I_H$$

Stage 2: Alternating iterations

For $k = 0, 1, \ldots, K$

(2.1) Updating the estimated signal

$$(\mathbf{d}^{k+1}, \mathbf{S}^{k+1}) = \underset{\mathbf{d},\mathbf{S}}{\operatorname{argmin}} \|A\mathbf{u}^k - \mathbf{d}\|^2 + \lambda_d \left\|\mathbf{d} - \tilde{\mathbf{d}}\right\|^2 + \lambda_d \lambda_{pd} \sum_{i_1,i_2,i_3} \left\| P_{(i_1,i_2,i_3)}(\tilde{\mathbf{d}}) - D\mathbf{S}_{(i_1,i_2,i_3)} \right\|^2$$

$$+ \lambda_d \lambda_{pd} \lambda_{sd} \sum_{i_1,i_2,i_3} \|P_{(i_1,i_2,i_3)}(\mathbf{d}) - D\mathbf{S}_{(i_1,i_2,i_3)}\|^2 + \lambda_d \lambda_{pd} \sum_{i_1,i_2,i_3,j} \left(\frac{\sigma}{d_j^T P_{(i_1,i_2,i_3)}(A\mathbf{u}^k)} \mathbf{S}_{(i_1,i_2,i_3)}(j) \right)^2$$

(2.2) Updating the reconstructed target

$$(\mathbf{u}^{k+1}, \mathbf{L}^{k+1}) = \underset{\mathbf{u},\mathbf{L}}{\operatorname{argmin}} \|A\mathbf{u} - \mathbf{d}^{k+1}\|^2 + s_u \sum_{i_1,i_2,i_3} \mathbf{L}(i_1,i_2,i_3)$$

$$+ \lambda_u \sum_{i_1,i_2,i_3} \|B_{i_1,i_2,i_3}(\mathbf{L}) - D_s^k C_{i_1,i_2,i_3}^k (D_n^k)^T\|^2 + \lambda_d \lambda_{pd} \sum_{i_1,i_2,i_3,j} \left(\frac{\sigma}{d_j^T P_{(i_1,i_2,i_3)}(A\mathbf{u})} \mathbf{S}_{(i_1,i_2,i_3)}^{k+1}(j) \right)^2$$

$$s.t. \ \mathbf{L}(i_1,i_2,i_3) = \sqrt{\sum_{j=1}^{3} \mathbf{u}(i_1,i_2,i_3,j)^2}$$

(2.3) Updating the dictionary atoms

$$(D_s^{k+1}, D_n^{k+1}, C^{k+1}) = \underset{D_s,D_n,C}{\operatorname{argmin}} \sum_{i_1,i_2,i_3} \left(\|B_{i_1,i_2,i_3}(\mathbf{L}^{k+1}) - D_s C_{i_1,i_2,i_3} D_n^T\|^2 + \lambda_{pu}^2 |C_{i_1,i_2,i_3}|_0 \right)$$

End

Stage 3: Output results

The estimated signal is given by $\mathbf{d}^K$.

The final reconstruction is given by $\mathbf{u}^K$.

The dictionary that captures local structures of the target is given by D_s^K.

The dictionary that captures nonlocal correlations of the target is given by D_n^K.

Algorithm 2: Initializing the reconstructed target

$\mathbf{b}_0^0 = \mathbf{0}$

$\mathbf{u}_0^0 = \underset{\mathbf{u}}{\operatorname{argmin}} \| A\,\mathbf{u} - \mathbf{d}^0 \|^2$

For $j = 0, 1, \ldots, J-1$ **do**

$\mathbf{v}_{j+1}^0 = \underset{\mathbf{v}}{\operatorname{argmin}}\ s_u \sum_{i_1,i_2,i_3} \sqrt{\sum_{j=1}^{3} \mathbf{v}\,(i_1,i_2,i_3,j)^2} + \mu \| \mathbf{v} - \mathbf{u}_j^0 + \mathbf{b}_j^0 \|^2$

with its entries given by

$$\mathbf{v}_{j+1}^0(i_1,i_2,i_3,:) =$$

$$\max\left(0, 1 - \frac{s_u}{2\mu \| \mathbf{u}_j^0(i_1,i_2,i_3,:) - \mathbf{b}_j^0(i_1,i_2,i_3,:) \|}\right) (\mathbf{u}_j^0(i_1,i_2,i_3,:) - \mathbf{b}_j^0(i_1,i_2,i_3,:))$$

$\mathbf{u}_{j+1}^0 = \underset{\mathbf{u}}{\operatorname{argmin}} \| A\,\mathbf{u} - \mathbf{d}^0 \|^2 + \mu^0 \| \mathbf{v}_{j+1}^0 - \mathbf{u} + \mathbf{b}_j^0 \|^2$

$\mathbf{b}_{j+1}^0 = \mathbf{b}_j^0 + \mathbf{v}_{j+1}^0 - \mathbf{u}_{j+1}^0$

End

$\mathbf{u}^0 = \mathbf{v}_J^0$

Algorithm 3: Updating the estimated signal

$\mathbf{d}_0^{k+1} = \frac{1}{1+\lambda_d} A\,\mathbf{u}^k + \frac{\lambda_d}{1+\lambda_d} \tilde{\mathbf{d}}$

For $j = 0, 1, \ldots, J-1$ **do**

$(\mathbf{S}_{(i_1,i_2,i_3)})_{j+1}^{k+1} = \frac{|D^T P_{(i_1,i_2,i_3)}(A\,\mathbf{u}^k)|^2}{|D^T P_{(i_1,i_2,i_3)}(A\,\mathbf{u}^k)|^2 + \frac{\sigma^2}{1+\lambda_{sd}}} \circ \left(D^T P_{(i_1,i_2,i_3)}\left(\frac{1}{1+\lambda_{sd}} \tilde{\mathbf{d}} + \frac{\lambda_{sd}}{1+\lambda_{sd}} \mathbf{d}_j^{k+1}\right)\right)$

in which the symbols $\circ$ and $|\cdot|^2$ stands for elementwise multiplication and square.

$\mathbf{S}_{j+1}^{k+1} = [\mathbf{S}_{(1,1,1)}^{k+1}, \ldots, \mathbf{S}_{(I_1,I_2,I_3)}^{k+1}]$

$\mathbf{d}_{j+1}^{k+1} = \frac{1}{1+\lambda_d+\lambda_d\lambda_{pd}\lambda_{sd}} A\,\mathbf{u}^k + \frac{\lambda_d}{1+\lambda_d+\lambda_d\lambda_{pd}\lambda_{sd}} \tilde{\mathbf{d}} + \frac{\lambda_d\lambda_{pd}\lambda_{sd}}{1+\lambda_d+\lambda_d\lambda_{pd}\lambda_{sd}} P^*(D\mathbf{S}_{j+1}^{k+1})$

End

$\mathbf{d}^{k+1} = \mathbf{d}_J^{k+1},\ \mathbf{S}^{k+1} = \mathbf{S}_J^{k+1}$

Algorithm 4: Updating the reconstructed target

$\mathbf{b}_0^{k+1} = \mathbf{0}$

$\mathbf{u}_0^{k+1} = \mathbf{u}^k$

For $j = 0, 1, \ldots, J-1$ **do**

$$\mathbf{v}_{j+1}^{k+1} = \underset{\mathbf{v}}{\text{argmin}}\ s_u \sum_{i_1, i_2, i_3} \sqrt{\sum_{j=1}^{3} \mathbf{v}\ (i_1, i_2, i_3, j)^2} + \mu \| \mathbf{v} - \mathbf{u}_j^{k+1} + \mathbf{b}_j^{k+1} \|^2$$

with its entries given by

$$\mathbf{v}_{j+1}^{k+1}(i_1, i_2, i_3, :) =$$

$$\max\left(0, 1 - \frac{s_u}{2\mu \| \mathbf{u}_j^{k+1}(i_1, i_2, i_3, :) - \mathbf{b}_j^{k+1}(i_1, i_2, i_3, :) \|}\right)(\mathbf{u}_j^{k+1}(i_1, i_2, i_3, :) - \mathbf{b}_j^{k+1}(i_1, i_2, i_3, :))$$

$$\mathbf{u}_{j+1}^{k+1} = \underset{\mathbf{u}}{\text{argmin}} \| A\,\mathbf{u} - \mathbf{d}^{k+1} \|^2 + \lambda_u \| \mathbf{u} - \mathbf{u}_{enhanced}^k \|^2 + \mu \| \mathbf{v}_{j+1}^{k+1} - \mathbf{u} + \mathbf{b}_j^{k+1} \|^2$$

in which $\mathbf{u}_{enhanced}^k$ is computed with equation (S.8).

$\mathbf{b}_{j+1}^{k+1} = \mathbf{b}_j^{k+1} + \mathbf{v}_{j+1}^{k+1} - \mathbf{u}_{j+1}^{k+1}$

End

$\mathbf{u}^{k+1} = \mathbf{v}_J^{k+1}$

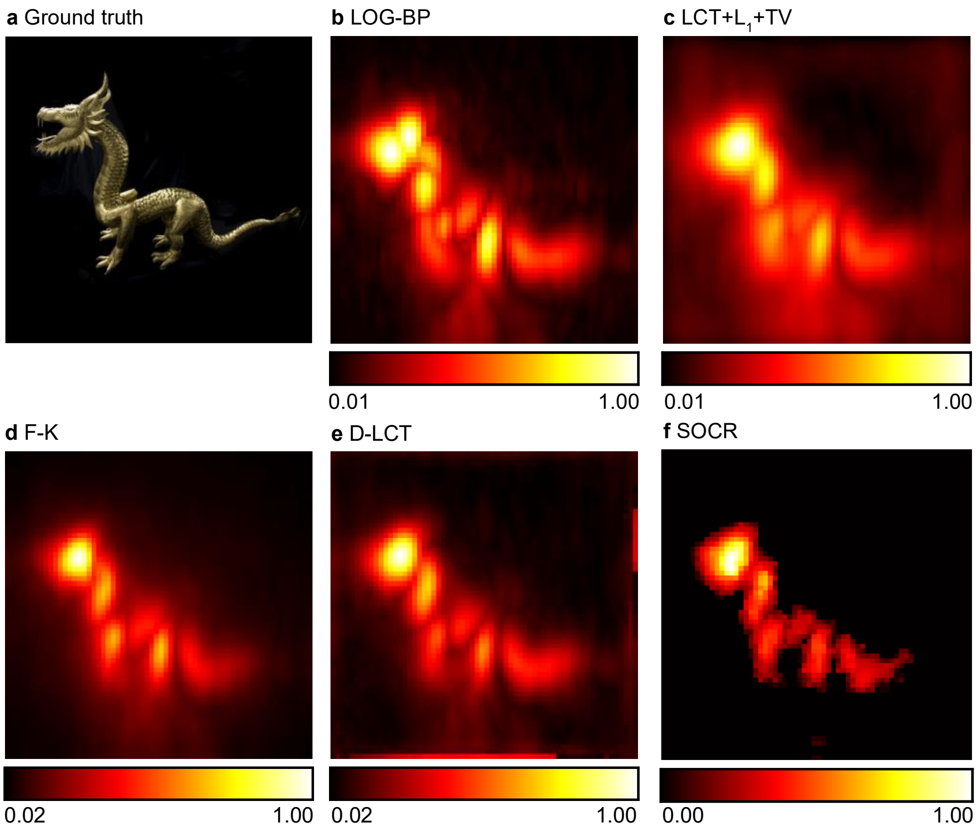

Fig. S1 Reconstruction results of the instance of the dragon with denoised measurement (confocal, 15 s). All methods fail to reconstruct the shape of the target precisely. The SOCR reconstruction has the highest contrast, with little noise in the background.

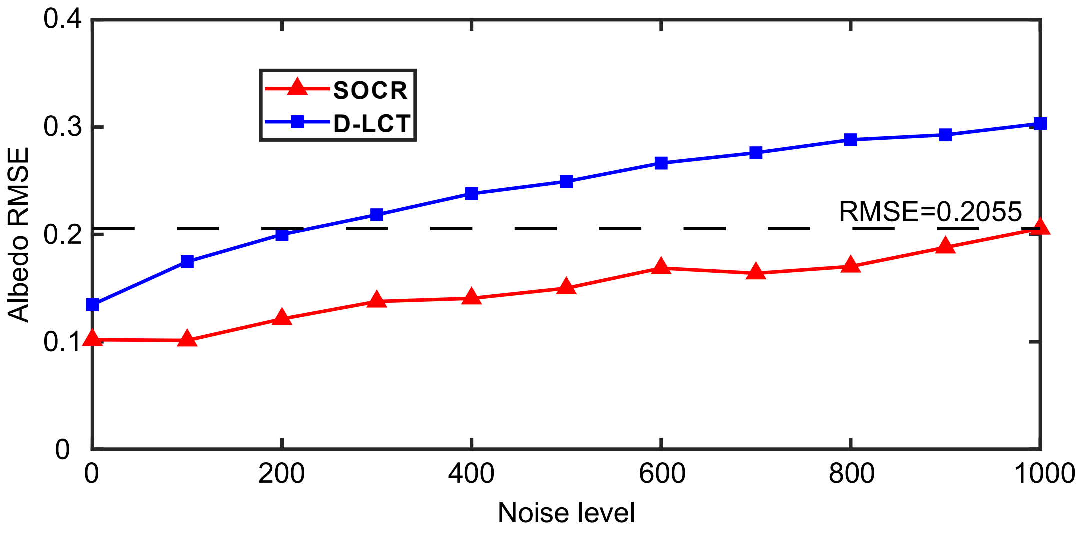

Fig. S2 Mean albedo RMSE of SOCR and D-LCT reconstructions under different noise levels. The SOCR reconstructions have smaller RMSE. When the noise level is 1000, the mean albedo RMSE of SOCR is 0.2055, which is smaller than that of D-LCT at noise level 300 (0.2183).

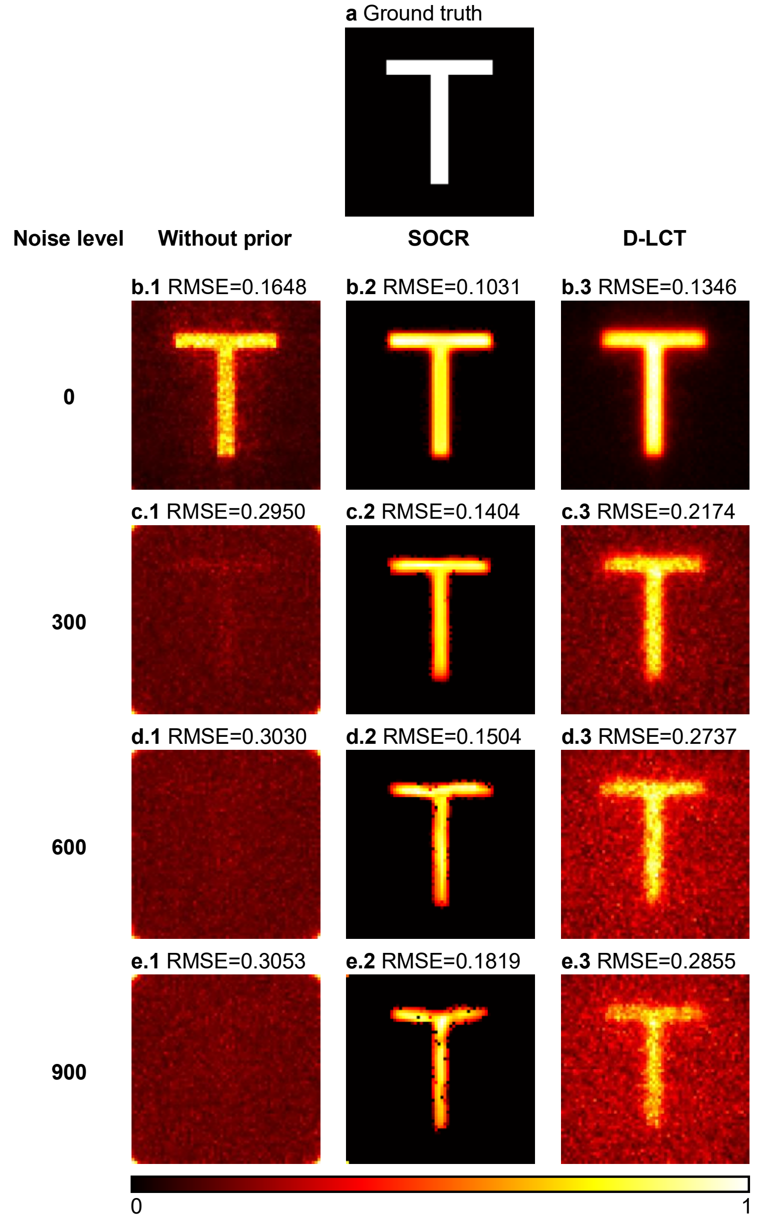

Fig. S3 Reconstruction results of the letter T under different noise levels. Reconstructions without regularization contain heavy background noise, and one can hardly identify the target when the noise level is high. The D-LCT method contains L_2 regularization and generates good results in low noise levels. The proposed SOCR method provides faithful reconstructions.
