# Supplementary figures and images for: Non-line-of-sight reconstruction with signal–object collaborative regularization
Source: Light Sci Appl. 2021 Sep 24;10:198. doi: 10.1038/s41377-021-00633-3 (PMC8463571; doi:10.1038/s41377-021-00633-3)

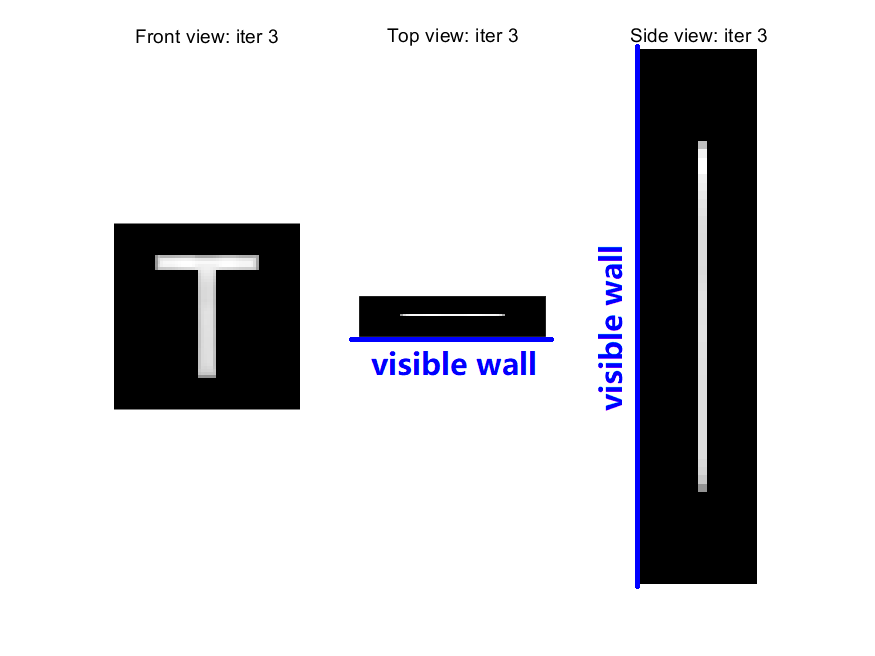

Supplement: Supplementary file 2 — Dataset [file 41377_2021_633_MOESM2_ESM.zip › NLOS_SOCR_code/read me.assets/three view after hard-thresholding.png]
